# Association Between REM Sleep Behavior Disorder and Cognitive Dysfunctions in Parkinson's Disease: A Systematic Review and Meta-Analysis of Observational Studies

**DOI:** 10.3389/fneur.2020.577874

**Published:** 2020-11-06

**Authors:** Jingrong Mao, Xiurong Huang, Jiaming Yu, Lang Chen, Yuqian Huang, Beisha Tang, Jifeng Guo

**Affiliations:** ^1^Department of Neurology, Xiangya Hospital, Central South University, Changsha, China; ^2^Xiangya School of Medicine, Central South University, Changsha, China; ^3^Zhongshan School of Medicine, Sun Yat-sen University, Guangzhou, China; ^4^Center for Inflammation and Epigenetics, Houston Methodist Research Institute, Houston, TX, United States; ^5^National Clinical Research Center for Geriatric Disorders, Xiangya Hospital, Central South University, Changsha, China

**Keywords:** rapid eye movement sleep behavior disorder (RBD), parkinson's disease (PD), dementia, meta-analysis, cognitive dysfunction

## Abstract

**Background:** Rapid eye movement sleep behavior disorder (RBD) is thought to be a prodromal symptom of Parkinson's disease (PD). RBD is also thought to be involved in cognitive decline and dementia in PD. In PD, although the relationship between RBD and cognitive dysfunctions was confirmed by considerable studies, whether RBD was associated with distinct types of cognitive defects is worth of study.

**Objectives:** This systematic review summarizes the evidence relating to cognitive dysfunction in PD patients with RBD (PD-RBD) and those without and explores their specificity to cognitive domains.

**Methods:** A meta-analysis using a random-effects model was performed for 16 different cognitive domains, including global cognitive function, memory (long-term verbal recall, long-term verbal recognition, long-term visual recall, short-term spatial recall, and short-term verbal recall), executive function (general, fluid reasoning, generativity, shifting, inhibition, and updating), language, processing speed/complex attention/working memory, visuospatial/constructional ability, and psychomotor ability. The cognitive difference between the groups of patients was measured as a standardized mean difference (SMD, Cohen's *d*). PD-RBD patients were classified into Confirmed-RBD (definite diagnosis with polysomnography, PSG) and Probable-RBD (without PSG re-confirmation). In some domains, RBD patients could not be analyzed separately due to the exiguity of primary studies; this analysis refers to such RBD patients as “Mixed-RBD.”

**Results:** Thirty-nine studies with 6,695 PD subjects were finally included. Confirmed-RBD patients showed worse performance than those without in global cognitive function, long-term verbal recall, long-term verbal recognition, generativity, inhibition, shifting, language, and visuospatial/constructional ability; Probable-RBD, in global cognitive function and shifting; and Mixed-RBD, in long-term visual recall, short-term spatial recall, general executive function, and processing speed/complex attention/working memory.

**Conclusion:** This meta-analysis strongly suggests a relationship between RBD, Confirmed-RBD in particular, and cognitive dysfunctions in PD patients. Early and routine screening by sensitive and targeted cognitive tasks is necessary for all PD-RBD patients because it may offer the therapeutic time window before they evolve to irreversible dementia.

## Introduction

Rapid eye movement sleep behavior disorder (RBD) is characterized by loss of the normal skeletal muscle atonia during rapid eye movement (REM) sleep, such that patients appear to act out the content of their dreams (1). Based on the third edition of the International Classification of Sleep Disorders (ICSD), the criteria for RBD were: (1) repeated episodes of behavior or vocalization that are either recorded by polysomnography (PSG) to arise from REM or are presumed to arise from REM based on reports of dream enactment and (2) evidence of REM sleep without atonia (RWA) on PSG (2). RBD has been linked to neurodegenerative pathology like Parkinson's disease (PD) (3). RBD was also identified as a key prodromal symptom of PD by the Movement Disorder Society (4). Approximately 75% of individuals who suffer from RBD progress to PD within 10 years (5, 6). Thus, the majority of patients manifesting RBD in sleep clinics are actually in the prodromal stages of PD.

Considerable studies suggested that RBD could be a key marker of a special subset of PD characterized by a non-tremor-dominant motor subtype or a kinetic-rigid motor phenotype (7–9) and symmetric disease (10, 11). RBD may also precede severe non-motor symptoms like increased autonomic dysfunction (12–15) and visual hallucinations (16–20). Of great interest, a concept— “RBD-PD phenotype” —was advanced. Poorer performance in memory, executive function (EF), and visuospatial abilities and a significantly greater risk of dementia were observed in PD patients who carry *GBA* gene mutations (21–23). *GBA* mutation carriers have a higher risk of developing probable RBD among PD patients (24). A study consisting 76 PD patients who were followed for an average of 4.5 years uncovered that the rate of deterioration was faster in the patients with RBD, mild cognitive impairment (MCI), and orthostatic hypotension at baseline (25). The similar conclusion that RBD is one of the most crucial prognosis determinants of PD was demonstrated by another follow-up study involving 421 PD patients for 32.8 ± 9.3 months (26). But two studies quarreled with this concept because neither significant gait disturbances and postural impairment nor specific worsening over time was observed in PD patients with RBD (27, 28).

Beyond these non-motor symptoms just mentioned, RBD usually predates, either by years or decades, the cognitive impairment or the diagnosis of MCI, which are the transitional states between normal aging and dementia in patients with PD (29, 30). Moreover, RBD increased the risk of cognitive decline and even dementia in PD patients (31–35). Considerable work reported an association between RBD and cognitive dysfunction and even dementia in PD [reviewed in (36)], but few suggested no significant declines in some targeted cognitive domains (37, 38) in PD patients with RBD.

The representative lineup includes RBD, “mild parkinsonian signs,” typical features of PD, PD with MCI, and a full dementia syndrome (PDD) (30), although not all of the patients follow this course of the disease. The endpoint of cognitive decline, PDD, is miserable and irreversible. These symptoms tend to appear in time intervals from months to years (39–41). The high conversion rate and the long latency of RBD to cognitive dysfunctions in PD patients make this study necessary and meaningful: cognitive dysfunction patients who suffer from both PD and RBD may be given opportunities for prevention and interventions before they progress to dementia.

Thus, this meta-analytic review was designed to shed light on the relationship between RBD and cognition in PD patients, as well as which cognitive domains are impaired. We also examined the influence of demographic and clinical confounders, like clinical stage, on cognitive performances in PD individuals.

## Materials and Methods

### Search Strategy

Consistent with PRISMA's suggestions (42), a systematic literature search was performed by two independent reviewers (JM and JY) up to 04 April 2020 using PsycInfo (PROQUEST), PubMed, Cochrane, and Embase. Searches were constructed using subtext headings and key words based on the following terms: RBD, cognitive impairment, and PD. For a detailed statement of the search, see Supplementary Table 1. The search was supplemented by hand searches of the reference lists cited in the original articles and review papers.

### Study Eligibility Criteria

#### Inclusion Criteria

This systematic review included studies investigating the effects of RBD in patients with PD on cognitive functions, published in peer-reviewed journals in English. Participants needed to be adults diagnosed with idiopathic PD based on any established international clinical criteria (43–47). RBD should ideally be diagnosed with PSG, while validated questionnaires or targeted interviews were also acceptable. The comparison had to be performed between parkinsonians with and without RBD (PD-RBD and PD-NRBD, respectively). Moreover, only studies assessing cognitive domains through standardized tests were included and the results had to be reported as the mean and standard deviation (SD) or the corresponding original data to allow the calculation of these values. When more than one study was published by the same authors, we checked the independence of samples or used the study with the largest sample size.

#### Exclusion Criteria

Proceedings, commentaries, letters to the editor, theses, studies performed on animals, and single case studies were all unacceptable. Studies that recruited atypical PD or parkinsonian syndromes were excluded. Cognition measured by subjective report or ratings-based methods or did not report the performance data for each cognitive task were discarded. Studies that concentrated on cognitive functions without linking them directly to RBD in PD or reported a comparison between PD patients and healthy participants were also unacceptable.

### Outcomes

For each study, the primary outcomes were cognitive test scores. Our main objective was to meta-analyze these scores to determine whether RBD in PD was associated with distinct types of cognitive defects. We categorized the cognitive tests following the approach described by Litvan et al. (48), Wallace and Bucks (49), and Olaithe and Bucks (50) or the indication provided in the primary studies. Subsequently, seven major cognitive categories were analyzed to organize the findings of this meta-analysis: global cognitive function, memory, EF, language, processing speed/complex attention/working memory, visuospatial/constructional ability, and psychomotor ability. Memory was further divided into long-term verbal recall, long-term verbal recognition, long-term visual recall, short-term spatial recall, and short-term verbal recall. EF was subdivided into the following: general EF, fluid reasoning, generativity, inhibition, shifting, and updating. Therefore, in total, 16 cognitive domains were compartmentalized, and the following analyses were carried out, respectively, in these segmentations. With several previous meta-analyses available for consultation (51–53) or the instructions of the included tests, we categorized the cognitive tests and listed them in Table 1.

**Table 1:** Cognitive domains and neuropsychological tests included in the primary studies.

**Categories**	**Tests**	**Included studies**
Global cognitive function	MMSE↑	Arnaldi et al. (54)^a^, Duarte Folle et al. (55)^a^, Ford et al. (56)^a^, Gagnon et al. (57)^a^, Gaudreault et al. (58)^a^, Gjerstad et al. (59)^a^, Huang et al. (60), Jozwiak et al. (5)^a^, Kamble et al. (61), Kim et al. (62)^a^, Kim et al. (63)^a^, Lavault et al. (28)^a^, Lee et al. (8)^a^, Lim et al. (64)^a^, Mahale et al. (65)^a^, Marques et al. (66)^a^, Naismith et al. (34)^a^, Nardone et al. (67)^a^, Nomura et al. (15)^a^, Nomura et al. (68)^a^, Nomura et al. (69), Nomura et al. (70)^a^, Plomhause et al. (71), Plomhause et al. (72), Postuma et al. (73), Rolinski et al. (33), Sinforiani et al. (19)^a^, Sixel-Doring et al. (16)^a^, Vendette et al. (35)^a^, Zhang et al. (74)
	MOCA↑	Ba et al. (75)^a^, Boucetta et al. (76)^a^, Chahine et al. (31)^a^, Huang et al. (60)^a^, Kamble et al. (61)^a^, Kotagal et al. (77)^a^, Liu et al. (78)^a^, Nomura et al. (69)^a^, Pagano et al. (79)^a^, Postuma et al. (73)^a^, Rahmani et al. (80)^a^, Rolinski et al. (33)^a^, Zhang et al. (74)^a^
	STMS↑	Meral et al. (81)^a^
	MDRS↑	Plomhause et al. (72)^a^
Long-term verbal recall	RAVLT, immediate recall↑	Gagnon et al. (82), Jozwiak et al. (5), Vendette et al. (35), Zhang et al. (74)
	RAVLT, delayed recall↑	Gagnon et al. (82)^a^, Jozwiak et al. (5)^a^, Vendette et al. (35)^a^, Zhang et al. (74)^a^
	RAVLT, list B↑	Gagnon et al. (82), Jozwiak et al. (5)
	RAVLT, sum of trials 1–5↑	Gagnon et al. (82), Jozwiak et al. (5), Vendette et al. (35)
	HVLT, immediate recall↑	Pagano et al. (79)^a^
	HVLT, delayed recall↑	Chahine et al. (31)^a^
	HVLT, total recall↑	Ba et al. (75)^a^
	SRT, immediate recall↑	Kamble et al. (61)
	SRT, delayed recall↑	Kamble et al. (61)^a^
	SBST, total recall↑	Meral et al. (81)
	SBST, delayed recall↑	Meral et al. (81)^a^
	Word list learning and recall test↑	Marques et al. (66)^a^, Sinforiani et al. (19)^a^
Long-term verbal recognition	RAVLT, recognition↑	Gagnon et al. (82)^a^, Jozwiak et al. (5)^a^, Vendette et al. (35)^a^, Zhang et al. (74)^a^
	HVLT, recognition↑	Ba et al. (75)^a^, Pagano et al. (79)^a^
	HVLT-R, recognition↑	Chahine et al. (31)^a^
	SBST, recognition↑	Meral et al. (81)^a^
Long-term visual recall	Wechsler memory scale↑	Meral et al. (81)^a^, Naismith et al. (34)^a^
	ROCF, immediate recall↑	Jozwiak et al. (5)
	ROCF, delayed recall↑	Jozwiak et al. (5)^a^, Zhang et al. (74)^a^
Short-term verbal recall	Digit span—forward↑	Kamble et al. (61)^a^, Marques et al. (66)^a^, Sinforiani et al. (19)^a^, Zhang et al. (74)^a^
Short-term spatial recall	CBTT↑	Kamble et al. (61)^a^, Sinforiani et al. (19)^a^
General executive function	FAB↑	Kamble et al. (61)^a^, Kim et al. (62)^a^, Lavault et al. (28)^a^, Sinforiani et al. (19)^a^
Fluid reasoning	Raven's progressive matrices↑	Sinforiani et al. (19)
Generativity	Verbal fluency—semantic↑	Ba et al. (75)^a^, Boucetta et al. (76)^a^, Chahine et al. (31)^a^, Gagnon et al. (82)^a^, Jozwiak et al. (5)^a^, Kamble et al. (61)^a^, Marques et al. (66)^a^, Meral et al. (81)^a^, Pagano et al. (79)^a^, Rolinski et al. (33)^a^, Vendette et al. (35)^a^, Zhang et al. (74)^a^
	Verbal fluency—letter↑	Chahine et al. (31), Gagnon et al. (82), Jozwiak et al. (5), Marques et al. (66), Rolinski et al. (33), Vendette et al. (35)
	Animal naming test↑	Kamble et al. (61)
Inhibition	Stroop task↓	Gagnon et al. (82)^a^, Jozwiak et al. (5)^a^, Kamble et al. (61)^a^, Marques et al. (66)^a^, Meral et al. (81)^a^, Vendette et al. (35)^a^, Zhang et al. (74)^a^
Shifting	TMT: B↓	Gagnon et al. (82)^a^, Jozwiak et al. (5)^a^, Vendette et al. (35)^a^, Zhang et al. (74)^a^
	TMT: B-A↓	Jozwiak et al. (5)
	WCST: Perseveration↓	Meral et al. (81)^a^, Sinforiani et al. (19)^a^
Updating	Digit span—backwards↑	Gagnon et al. (82)^a^, Jozwiak et al. (5)^a^, Kamble et al. (61)^a^, Marques et al. (66)^a^, Naismith et al. (34)^a^, Zhang et al. (74)^a^
Language	Boston naming test↑	Jozwiak et al. (5)^a^
	Lexis denomination task↑	Plomhause et al. (72)^a^
Processing speed/Complex attention/Working memory	LNS↑	Ba et al. (75)^a^, Chahine et al. (31)^a^, Marques et al. (66)^a^, Pagano et al. (79)^a^
	TMT: A↓	Jozwiak et al. (5)^a^, Vendette et al. (35)^a^, Zhang et al. (74)^a^
Visuospatial/Constructional ability	BJLOT↑	Ba et al. (75)^a^, Boucetta et al. (76)^a^, Chahine et al. (31)^a^, Meral et al. (81)^a^, Pagano et al. (79)^a^
	Clock drawing test↑	Meral et al. (81), Zhang et al. (74)
	ROCF, copy↑	Gagnon et al. (82)^a^, Jozwiak et al. (5)^a^, Vendette et al. (35)^a^, Zhang et al. (74)^a^
	Bells test↓	Gagnon et al. (82), Vendette et al. (35)
	Block design↑	Gagnon et al. (82), Vendette et al. (35)
	BFRT↑	Meral et al. (81)
Psychomotor ability	Symbol digit modalities↑	Ba et al. (75)^a^, Boucetta et al. (76)^a^, Chahine et al. (31)^a^, Marques et al. (66)^a^, Pagano et al. (79)^a^, Zhang et al. (74)^a^

↑ *Better performance with higher scores*; ↓ *Worse performance with higher scores*.

*MMSE, Mini-Mental State Examination; MOCA, Montreal Cognitive Assessment; STMS, Short Test of Mental Status; MDRS, Mattis Dementia Rating Scale; RAVLT, Rey Auditory–Verbal Learning Test; HVLT, Hopkins Verbal Learning Test; SRT, story recall test; SBST, sözel bellek surecleri testi; HVLT-R, Hopkins Verbal Learning Test—Revised; ROCF, Rey–Osterrieth complex figure test; CBTT, Corsi's block tapping test; FAB, frontal assessment battery; TMT: B, trail making test: part B; TMT: B-A, trail making test: part B–part A; WCST, Wisconsin Card Sorting Test; LNS, letter–number sequencing; TMT: A, trail making test: part A; BJLOT, Benton judgment of line orientation test; BFRT, Benton's face recognition test*.

a *Test is analyzed in a meta-analysis*.

Cognitive domains assessed by only one study could not be included. When a cognitive function was explored by more than one test in a primary study, two different strategies were adopted by previous meta-analyses: some extracted data from the most sensitive and relevant instrument (52, 53), while some aggregated the results into a single effect size (ES) (83–85). These two strategies are both valid and have their own advantages; the first solution diminishes the risk of type II errors, while the second strategy decreases bias of a certain test. We decided to follow the first solution. As for the criteria for the “most sensitive and relevant instrument,” the sensitivity and relevance in the PD population of each test were checked on the basis of already-published research first, and preference was given to the highest sensitivity and/or relevant test if more than one test assessing the same cognitive domain were adopted in a primary study. If the sensitivity and/or relevance was not available, the most used test was analyzed in this domain. Thus, sensitivity, relevance, and popularity, in this order, are what we considered in choosing assessable tests. This criterion was consistently used across all domains in this meta-analysis.

### Data Extraction and Coding

Data extracted and coded from the primary studies included: (1) characters of the publication (e.g., authors and year of publication); (2) diagnoses of PD and RBD; (3) characteristics of the sample [e.g., sample size, gender, age at evaluation, disease duration, education, severity of motor symptoms evaluated by the Unified Parkinson's Disease Rating Scale (UPDRS-III), stage of PD evaluated by Hoehn and Yahr (H&Y), and the levodopa equivalent daily dose (LEDD)]; and (4) cognitive tests.

### Statistical Analysis

All statistical analyses were performed using RevMan 5.3 (The Nordic Cochrane Centre, The Cochrane Collaboration, Copenhagen, Denmark) and Stata/SE version 15 (StataCorp, College Station, TX, USA).

SMD was used as the outcome measure because, although the included studies all assessed the same cognitive function within one meta-analysis, different cognitive tests were employed. ESs were categorized using Cohen's *d* as 0.2, indicating a small effect, 0.5, a medium, and 0.8, large. When calculating ESs, the PD-NRBD scores were always subtracted from the PD-RBD scores. Cognitive tests broadly fit into two categories: one where higher scores indicate better performance, namely milder damage, and the other where higher scores conversely represent greater impairment. A negative ES in the former tests indicates that the PD-RBD participants were more impaired than the PD-NRBD participants, as opposed to the latter cases. Random-effects models were applied to all cognitive domains.

The methodological quality of the enrolled cohort and case–control studies was evaluated with the Newcastle–Ottawa Scale (86) and the cross-sectional studies with the modified Newcastle–Ottawa Scale (87). Reports that scored ≥6 points were considered to be of good quality. The quality assessment was performed independently by two authors (JM and JY) and disagreements were resolved by discussion.

Prior defined subgroup analyses were performed based on whether the diagnosis of RBD was confirmed by PSG. Hence, studies were placed in the “Confirmed-RBD” subgroup if the RBD patients met the ICSD criteria where PSG is mandatory. Conversely, studies were placed in the “Probable-RBD” subgroup if the diagnosis was made by questionnaires and/or interviews. In some domains, RBD patients could not be analyzed separately due to the exiguity of the primary studies in which they were enrolled, and they were referred to as “Mixed-RBD.” Concretely, in a certain domain, only one primary study used PSG to confirm RBD; therefore, patients from this study were Confirmed-RBD patients. Meanwhile, the patients included in the other studies were Probable-RBD patients not confirmed by PSG. Since one study could not be meta-analyzed, we combined and analyzed the results of all RBD patients, for both Confirmed-RBD and Probable-RBD, denoted as “Mixed-RBD.”

Another subgroup analysis was performed considering the possible differential effects of clonazepam, the major treatment for RBD, which may deteriorate cognitive dysfunctions (88–90). Therefore, studies were placed in either the “Mediated by Clonazepam” subgroup if medicated patients were recruited or “Unmediated by Clonazepam” subgroup when mediated patients were excluded or the dose they were taking was negligible.

Meta-regressions were performed to investigate whether the outcomes were affected by other characteristics, including demographic characteristics (age at evaluation, gender, and education), severity of PD (PD duration, UPDRS-III, H&Y stage, and LEDD), cognitive tests, and tools used to assess RBD. These covariates were meta-regressed individually in a random-effects meta-regression model. According to Borenstein et al. (91), a meta-regression could be generally conducted for outcomes where there are 10 samples at a minimum to one covariate. But given that the majority of domains included <10 reports, with reference to Taylor et al. (92), we liberalized the restriction to five.

The heterogeneity test was quantified using the *I*^2^ statistic. The *I*^2^ was set as low (25%), moderate (50%), or high (75%). Sensitivity analysis was conducted for meta-analysis where *I*^2^ ≥ 50% by omitting the enrolled studies, one at a time, to determine the effect of any individual study on the synthesized ES and between-study heterogeneity. Finally, publication bias analysis was performed with the funnel plot of which the asymmetry was further statistically confirmed by the Egger's regression method and the trim-and-fill procedure in the meta-analyses that included ≥10 studies.

All statistical tests were two-tailed, and *P* < 0.05 was considered significant.

## Results

### Study Selection and Risk of Bias

**Figure 1:**
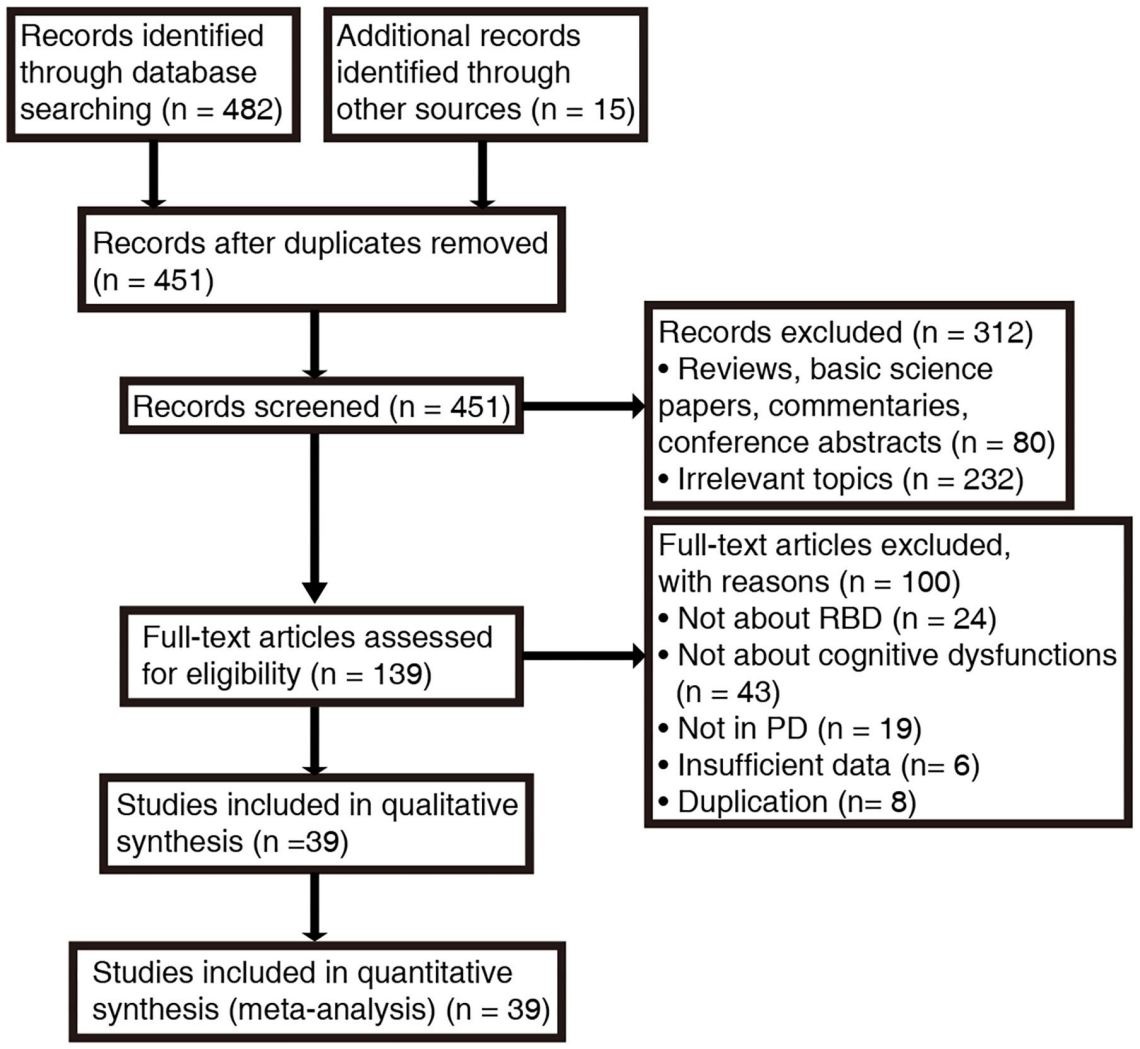
Search procedure and results according to PRISMA guidelines. *RBD*, rapid eye movement sleep behavior disorder; *PD*, Parkinson's disease.

A total of 482 papers were produced according to our search strategy, and 15 additional records were identified from the references cited in the original articles and review papers. Following exclusion of duplicates and unrelated studies based on title and abstract screening, we retrieved 139 papers for full-text evaluation. The PRISMA flow diagram (Figure 1) summarizes the selection process. In total, 39 studies were enrolled after rigorous screening (5, 8, 15, 16, 19, 28, 31, 33–35, 54–82). A critical appraisal assessment found that all studies exhibited “good quality,” with the score ranging from 6 to 9, and no studies were excluded due to quality issues (Supplementary Tables 2, 3).

### Characteristics of the Included Studies

The characteristics of the included studies are summarized in Table 2. Across these 39 studies, 6,695 individuals with PD were investigated, with the mean age ranging from 57.3 to 76.5 years. The mean UPDRS-III scores were provided in 30 studies, while either the mean or median H&Y stage values were reported in 25. Two studies (63, 66) did not provide either measure of motor symptoms or disease stage for the entire sample. PSG was used in 18 studies alone or combined with the clinical interview (5, 15, 16, 35, 54, 57, 58, 60, 61, 63, 66–68, 70–73, 82). The remainder adopted sleep questionnaires and/or clinical interviews to identify Probable-RBD. Ten studies (16, 33, 57–59, 61, 69, 70, 78, 82) particularly pointed out the utilization of clonazepam.

**Table 2:** Characteristics of the primary studies included in the meta-analysis.

**Study**	**Diagnostic criteria for PD**	**RBD assessment**	**Participants (*n* men)**	**Age (years)**	**PD duration (years)**	**Edu**	**UPDRS-III**	**H&Y stage**	**LEDD (mg)**	**Analyzed cognitive tests**
**CONFIRMED-RBD**										
Arnaldi et al. (54)	Clinical criteria Gelb et al. (43)	PSG + clinical evaluation (ICSD-2)	24 RBD+ (16)	69.4 ± 6.0	NM	10.3 ± 4.6	14.1 ± 5.7	NM	0	MMSE
			16 RBD– (8)	67.2 ± 7.2	NM	11.8 ± 5.0	9.5 ± 3.5	NM	0	
Gagnon et al. (57)	NM	PSG	7 RBD+ (6)	68.4 ± 7.5	5.4 ± 6.0	NM	NM	1.8 ± 0.8	313.6 ± 184.8	MMSE
			8 RBD– (3)	61.0 ± 7.3	5.5 ± 2.9	NM	NM	1.8 ± 0.7	277.3 ± 148.0	
Gagnon et al. (82)	PDSBB	PSG (ICSD-2)	22 RBD+ 18 RBD–	66.4 ± 8.5 65.2 ± 8.9	4.9 ± 3.5 5.8 ± 3.2	14.7 ± 3.9 15.4 ± 2.1	18.6 ± 7.7 17.3 ± 11.0	2.0 ± 0.8 2.1 ± 0.8	392.6 ± 363.6 353.6 ± 324.4	RAVLT, delayed recall; RAVLT, recognition; SVF; DSB; TMT: B; Stroop errors; ROCF, copy
Gaudreault et al. (58)	PDSBB	PSG (ICSD-2)	16 RBD+ (11)	64.7 ± 8.0	5.4 ± 3.5	14.8 ± 4.1	18.1 ± 8.6	2.1 ± 0.8	506.1 ± 383.3	MMSE
			15 RBD– (9)	63.1 ± 6.0	5.4 ± 3.8	14.5 ± 3.3	20.7 ± 9.7	2.2 ± 0.8	398.4 ± 273.7	
Huang et al. (60)	PDSBB	PSG + clinical evaluation (ICSD-3)	92 RBD+ (68)	65.1 ± 5.8	3.0 (1.0–5.0)^b^	8.4 ± 3.5	24.3 ± 10.0	2.0 (1.5–2.5)^b^	300.0 (100.0–450.0)^b^	MOCA
			82 RBD– (45)	64.0 ± 9.3	3.0 (1.0–5.0)^b^	8.8 ± 3.2	23.7 ± 11.5	2.0 (1.5–2.5)^b^	300.0 (100.0–450.0)^b^	
Jozwiak et al. (5)	NM	PSG (ICSD-2)	53 RBD+ (40) 40 RBD– (21)	68.0 ± 8.4 63.2 ± 8.5	6.1 ± 4.5 6.1 ± 4.3	14.5 ± 3.9 15.1 ± 3.0	23.1 ± 9.5 20.3 ± 9.8	2.5 ± 0.8 2.2 ± 0.9	492.2 ± 402.3 383.7 ± 283.3	MMSE; vocabulary; RAVLT, delayed recall; RAVLT, recognition; ROCF, delayed recall; SVF; TMT: B; Stroop errors; TMT: A; ROCF, Copy
Kamble et al. (61)	PDSBB	MSQ + RBDSQ + PSG	25 RBD+	60.4 ± 8.2	6.8 ± 4.6	NM	27.4 ± 11.1	NM	535 ± 178.9	MOCA; story recall (delayed); CBTT; FAB; DSB; SVF; DSF
			25 RBD–	57.3 ± 6.6	7.5 ± 3.5	NM	32.7 ± 8.22	NM	754 ± 349.7	
Kim et al. (63)	PDSBB	PSG + RBDSQ-K	9 RBD+ (0)	70.1 ± 6.8	1.9 ± 1.5	2.4 ± 2.5	NM	NM	NM	K-MMSE
			22 RBD– (10)	67.7 ± 8.4	1.9 ± 1.4	8.5 ± 4.8	NM	NM	NM	
Marques et al. (66)	Clinical criteria Gelb et al. (43)	PSG	10 RBD+ (3) 10 RBD– (3)	64 ± 2.9 59 ± 2.6	7.6 ± 1.7 8.1 ± 3.7	10 ± 0.6 10 ± 0.9	NM NM	NM NM	703 ± 157 435 ± 133	MMSE; Number of words correctly encoded; DSB; LNS; Stroop color-word; SVF; SDMT; DSF
Nardone et al. (67)	NM	PSG + clinical evaluation (ICSD-2)	10 RBD+ (8)	65.9 ± 6.5	5.0 ± 2.3	10.1 ± 3.8	17.5 ± 4.3	NM	578 ± 2.9	MMSE
			13 RBD– (9)	63.7 ± 6.4	6.0 ± 2.8	10.5 ± 3.7	18.3 ± 4.3	NM	627 ± 341	
Nomura et al. (15)	NM	PSG + interview	18 RBD+ (5)	71.3 ± 8.3	9.0 ± 4.7	NM	NM	3.0 ± 0.9	408 ± 214	MMSE
			23 RBD– (10)	71.5 ± 7.2	5.3 ± 4.8	NM	NM	2.7 ± 0.9	347 ± 199	
Nomura et al. (68)	PDSBB	PSG + interview	27 RBD+ (14)	76.5 ± 5.9	8.8 ± 5.2	NM	NM	3.0 ± 0.8	455 ± 230	MMSE
			32 RBD– (14)	74.5 ± 8.1	7.0 ± 8.2	NM	NM	2.5 ± 0.6	233 ± 150	
Nomura et al. (70)	PDSBB	PSG + clinical evaluation (ICSD-3)	47 RBD+ (26)	73.1 ± 7.3	9.0 ± 6.0	NM	NM	3.0 ± 0.8	437 ± 250	MMSE
			89 RBD– (35)	71.1 ± 7.8	5.7 ± 6.8	NM	NM	2.5 ± 0.7	250 ± 199	
Plomhause et al. (71)	Clinical criteria Gibb and Lees (45)	PSG + clinical evaluation (ICSD-2)	17 RBD+ (8)	65 ± 8	11 ± 4^c^	NM	14 ± 8	NM	0	MDRS
			40 RBD– (27)	60 ± 12	15 ± 12^c^	NM	15 ± 6	NM	0	
Plomhause et al. (72)	Clinical criteria Gelb et al. (43)	PSG + clinical evaluation (ICSD-2)	15 RBD+ (14)	63.2 ± 7.7	7.0 ± 3.7	11.8 ± 3.9	18.8 ± 8.6	NM	435.1 ± 171.1	MDRS; Lexis denomination task
			15 RBD– (11)	61.4 ± 7.5	4.1 ± 3.2	10.5 ± 1.7	19.8 ± 6.5	NM	633.0 ± 342.3	
Postuma et al. (73)	PDSBB	PSG + clinical evaluation (ICSD-2)	27 RBD+ (23)	70.5 ± 7.4	9.7 ± 4.3	NM	34.1 ± 16.5	NM	NM	MOCA
			15 RBD– (11)	67.5 ± 10.5	9.5 ± 4.8	NM	26.2 ± 16.2	NM	NM	
Sixel-Doring et al. (16)	PDSBB	PSG	210 RBD+ (62)	69 ± 8	8.7 ± 4.4	5.2 ± 4.6	30 ± 14	3.2 ± 1.1	500.6 ± 375.3	MMSE
			247 RBD– (64)	66 ± 11	7.3 ± 5.6	4.2 ± 3.6	28 ± 15	2.9 ± 0.9	422.9 ± 330.4	
Vendette et al. (35)	PDSBB	PSG + clinical evaluation (ICSD-2)	18 RBD+ 16 RBD–	65.61 ± 7.73 65.13 ± 7.69	5.2 ± 2.3 6.0 ± 3.2	15.0 ± 3.7 15.8 ± 1.9	17.3 ± 7.7Δ 15.6 ± 10.7Δ	2.06 ± 0.78 2.22 ± 0.77	417.2 ± 425.6 399.8 ± 315.0	MMSE; RAVLT, delayed recall; RAVLT, recognition; SVF; TMT: B; Stroop errors; TMT: A; ROCF, Copy
**PROBABLE-RBD**										
Ba et al. (75)	Clinical criteria^a^ + DAT imaging deficit	RBDSQ (>5)	136 RBD+ (92) 214 RBD– (137)	61.2 ± 9.4 60.4 ± 9.9	7.51 ± 6.69^c^ 7.35 ± 6.29^c^	15.6 ± 2.9 15.6 ± 3.0	21.6 ± 9.4 19.6 ± 8.4	1.6 ± 0.5 1.5 ± 0.5	0 0	MOCA; HVLT, total recall; HVLT, Recognition; SVF; SDMT; LNS; BJLOT
Boucetta et al. (76)	Clinical criteria^a^	RBDSQ (≥5) + positive response to item 5, 6.3 or 6.4	69 RBD+ (52) 240 RBD– (149)	60.9 ± 9.2 61.6 ± 9.8	6.3 ± 6.6^c^ 6.9 ± 6.7^c^	15.9 ± 2.3 15.6 ± 2.9	22.5 ± 9.5	NM NM	0 0	MOCA; SVF; SDMT; BJLOT
Chahine et al. (31)	Clinical criteria^a^ + DAT imaging deficit	RBDSQ (≥6)	108 RBD+ (79) 315 RBD– (198)	61.9 ± 9.9 61.7 ± 9.7	0.25 (0.17–0.59)^b^ 0.34 (0.25–0.67)^b^	15.3 ± 2.9 15.6 ± 3.0	22.0 ± 8.8 20.5 ± 8.9	NM NM	0 0	MOCA; HVLT-R, delayed free recall; HVLT-R, recognition; SVF; SDMT; LNS; BJLOT
Duarte Folle et al. (55)	NM	Interview	160 RBD+ (122)	70.0 ± 9.6	3.5 ± 3.1	14.3 ± 3.7	23.4 ± 2.5	≥3, N = 25	459 ± 349	MMSE
			616 RBD– (371)	70.6 ± 10.4	3 ± 2.5	13.6 ± 4.7	22.6 ± 2.4	≥3, N = 99	388 ± 332	
Ford et al. (56)	PDSBB	MSQ	46 RBD+ (36)	66.4 ± 9.9	6.5 ± 5.1^c^	13.0 ± 3.6	26.3 ± 10.0	2.2 ± 0.7	179.2 ± 144.7	MMSE
			78 RBD– (48)	65.8 ± 10.9	6.0 ± 4.4^c^	13.0 ± 4.1	27.3 ± 11.9	1.9 ± 0.6	172.6 ± 128.2	
Gjerstad et al. (59)	Clinical criteria (93)	SSQ	34 RBD+ (25)	71.6 ± 7.9	11.1 ± 6.2	NM	29.5 ± 15.8	3.0 ± 1.1	626 ± 312	MMSE
			197 RBD– (89)	73.7 ± 8.5	8.6 ± 5.5	NM	28.2 ± 15.9	2.8 ± 1.0	452 ± 236	
Kim et al. (62)	PDSBB	ICSD-R	578 RBD+ (266)	64.6 ± 8.8	7.66 ± 4.66	NM	NM	2.25^b^	795.3 ± 406.2	MMSE; FAB
			366 RBD– (182)	62.2 ± 10.0	6.21 ± 3.91	NM	NM	2.03^b^	693.0 ± 421.6	
Kotagal et al. (77)	PDSBB + DTBZ PET imaging	MSQ	27 RBD+ (25)	63.4 ± 6.7	6.4 ± 3.7	NM	27.6 ± 10.9	2.3 ± 0.4	NM	MOCA
			53 RBD– (35)	65.3 ± 7.1	5.8 ± 4.0	NM	25.1 ± 11.2	2.3 ± 0.5	NM	
Lavault et al. (28)	PDSBB	Interview	39 RBD+ (26)	66.6 ± 7.9	7.5 ± 5.1	NM	22.0 ± 12.4	NM	576 ± 353	MMSE; FAB
			22 RBD– (13)	60.3 ± 11.1	5.8 ± 3.9	NM	13.5 ± 6.5	NM	649 ± 403	
Lee et al. (8)	PDSBB	ICSD-R	164 RBD+ (79)	65.1 ± 8.4	7.28 ± 5.18	NM	20.3 ± 10.4	2.2 ± 0.7	527 ± 292	MMSE
			283 RBD– (128)	63.1 ± 9.6	5.47 ± 4.16	NM	18.2 ± 11.2	2.1 ± 0.6	485 ± 285	
Lim et al. (64)	PDSBB	RBDSQ + PSG (partial confirmation)	24 RBD+ (12)	69.8 ± 6.4	6.2 ± 2.9	NM	12.4 ± 2.5	1.9 ± 0.4	NM	MMSE
			14 RBD– (8)	69.7 ± 7.2	4.4 ± 3.7	NM	22.4 ± 10.6	1.6 ± 0.5	NM	
Liu et al. (78)	PDSBB	RBDSQ (≥5)	31 RBD+ (14)	60.4 ± 10.8	2.71 ± 3.52	9.2 ± 3.8	21.3 ± 9.0	1.9 ± 0.5	0	MOCA
			127 RBD– (61)	58.3 ± 10.4	1.90 ± 1.72	10.3 ± 3.9	20.1 ± 10.9	2.0 ± 0.4	0	
Mahale et al. (65)	Queen Square Brain Bank criteria	RBDSQ	10 RBD+ (0)	62.5 ± 10.3	4.1 ± 2.8	NM	27.5 ± 9.9	2.4^b^	675.0 ± 521.5	MMSE
			27 RBD– (0)	51.2 ± 10.7	5.3 ± 4.9	NM	31.0 ± 9.7	2.3^b^	588.5 ± 313.5	
Meral et al. (81)	PDSBB	Interview (ICSD)	36 RBD+ (24) 43 RBD– (26)	66.5 ± 8.79 67.6 ± 8.5	7.27 ± 3.59 5.60 ± 3.65	NM NM	23.3 ± 11.5 17.5 ± 11.0	NA NA	423.2 ± 198.8 339.0 ± 245.9	STMS; WMS, delayed recall; SBST, recognition; SBST, delayed recall; SVF; stroop error; WCST, category; BJLOT
Naismith et al. (34)	PDSBB	RBDSQ (≥5)	51 RBD+ 47 RBD–	65.5 ± 7.2 65.0 ± 9.7	6.8 ± 5.7 4.6 ± 4.4	13.3 ± 2.9 14.2 ± 3.3	NM NM	2.2 ± 0.7 2.2 ± 0.7	773.3 ± 577.7 512.9 ± 551.1	MMSE; Logical memory, encoding; DSB; TMT: B-A
Nomura et al. (69)	PDSBB	RBDSQ-J (≥6)	27 RBD+ (15)	68.8 ± 8.6	9.1 ± 7.7	NM	NM	2.8 ± 0.7	500 ± 351	MOCA
			43 RBD– (16)	69.4 ± 8.6	6.4 ± 5.2	NM	NM	2.5 ± 0.9	322 ± 243	
Pagano et al. (79)	Clinical criteria^a^ + DAT imaging deficit	RBDSQ (≥5)	158 RBD+ (109) 263 RBD– (166)	61.8 ± 9.7 61.5 ± 9.8	6.5 ± 6.5^c^ 6.7 ± 6.6^c^	15.6 ± 2.9 15.5 ± 3.0	20.9 ± 8.8 20.4 ± 8.8	1.54 ± 0.50 1.58 ± 0.51	0	MOCA; HVLT, immediate recall; HVLT, delayed recognition; SVF; LNS; SDMT; BJLOT
Rahmani et al. (80)	Clinical criteria^a^	RBDSQ	10 RBD+ (8)	61 ± 8.15	NM	15.8 ± 3.1	18.9 ± 10.8	NM	NM	MOCA
			7 RBD– (6)	64 ± 6.6	NM	14.4 ± 3.4	24.5 ± 6.9	NM	NM	
Rolinski et al. (33)	PDSBB	RBDSQ	224 RBD+ (148)	67.5 ± 9.4	1.6 ± 1.0	NM	26.8 ± 10.6	1.9 ± 0.5	345.2 ± 189.6	MOCA; SVF
			251 RBD– (144)	67.9 ± 9.5	1.4 ± 1.0	NM	26.9 ± 11.2	1.9 ± 0.5	322.9 ± 196.7	
Sinforiani et al. (19)	PDSBB	Clinical evaluation (ICSD)	79 RBD+ (46) 31 RBD– (19)	68.0 ± 8.4 63.0 ± 8.2	10.3 ± 4.9 9.5 ± 4.5	NM NM	47.7 ± 12.6 35.2 ± 10.3	3.5^b^ 3^b^	1087.9 ± 442.3 1114.5 ± 270.4	MMSE; CBTT; DSF; Logical memory test; FAB
Zhang et al. (74)	PDSBB	MSQ + RBDSQ + PSG (partial confirmation)	32 RBD+ (23) 42 RBD– (20)	64.9 ± 5.2 62.2 ± 8.3	4.0 ± 2.5 4.2 ± 2.7	9.5 ± 2.3 9.8 ± 2.6	19.8 ± 12.0 20.0 ± 9.6	2.0 (1.5–2.5)^b^ 2.0 (1.5–2.5)^b^	292.3 ± 210.2 298.2 ± 237.2	MOCA; SVF; RAVLT, delayed recall; RAVLT, recognition; ROCF, recall; DSB; TMT: B, time; Stroop time; DSF; TMT: A; SDMT; ROCF, Copy

*Data are shown as the mean ± SD unless otherwise noted*.

*PD, Parkinson's disease; RBD, REM sleep behavior disorder; Edu, education; UPDRS III, Unified Parkinson's Disease Rating Scale, part 3; H&Y stage, Hoehn and Yahr stage; LEDD, levodopa equivalent daily dose; PSG, polysomnography; ICSD-2, second edition of the International Classification of Sleep Disorders; NM, not mentioned; MMSE, Mini-Mental State Examination; PDSBB, U.K. Parkinson's Disease Society Brain Bank criteria; RAVLT, Rey auditory–verbal learning test; SVF, semantic/category verbal fluency test; DSB, digit span backward; TMT: B, trail making test: part B; ROCF, Rey–Osterrieth complex figure test; ICSD-3, third edition of the International Classification of Sleep Disorders; MOCA, Montreal Cognitive Assessment; TMT: A, trail making test: part A; MSQ, Mayo Sleep Questionnaire; RBDSQ, REM Sleep Behavior Disorder Screening Questionnaire; CBTT, Corsi's block tapping test; FAB, frontal assessment battery; DSF, digit span forward; RBDSQ-K, Korean version of REM Sleep Behavior Disorder Screening Questionnaire; K-MMSE, Korean version of the Mini-Mental State Examination; LNS, letter–number sequencing; SDMT, symbol digit modalities test; MDRS, Mattis Dementia Rating Scale; DAT, dopamine transporter; HVLT, Hopkins Verbal Learning Test; BJLOT, Benton judgment of line orientation test; HVLT-R, Hopkins Verbal Learning Test—Revised; SSQ, Stavanger Sleepiness Questionnaire; ICSD-R, International Classification of Sleep Disorders—Revised; DTBZ, dopaminergic denervation on [^11^C]dihydrotetrabenazine; STMS, Short Test of Mental Status; WMS, Wechsler Memory Scale; SBST, sözel bellek surecleri testi; WCST, Wisconsin Card Sorting Test; TMT: B-A, trail making test: part B–part A; RBDSQ-J, Japanese version of REM Sleep Behavior Disorder Screening Questionnaire*.

a *All clinical data were extracted from the PPMI database. Further information is described in detail at http://www.ppmi-info.org*.

b *Median (IQR)*.

c *Months*.

Some reports (5, 35, 54, 58, 64, 66, 72, 74, 76, 82) also recruited idiopathic RBD patients and/or health controls in addition to our targeted individuals and did comparisons between any two, but we only extracted the statistics from PD-RBD and PD-NRBD patients. Moreover, PD-RBD patients were further classified into subgroups in four studies: clinical or subclinical PD-RBD (15, 68) and PD-RBD with and without visual hallucinations (VH) (19, 81). In the former two studies (15, 68), patients with both RWA on PSG and RBD symptoms were classified as the “RBD group”; patients who only manifested RWA without RBD symptoms were categorized as the “Subclinical RBD group.” Given that the rest of the reports did not recruit or subgroup the subclinical RBD patients, we only extracted data from the “RBD group” and the “NRBD group” and dropped the data from the “Subclinical RBD group.” The latter two studies (19, 81) examined VH besides RBD. Because VH was not the outcome of interest of this review, the groups were collapsed into RBD+ and RBD–.

### Meta-Analytic Results

#### Global Cognitive Function

**Figure 2:**
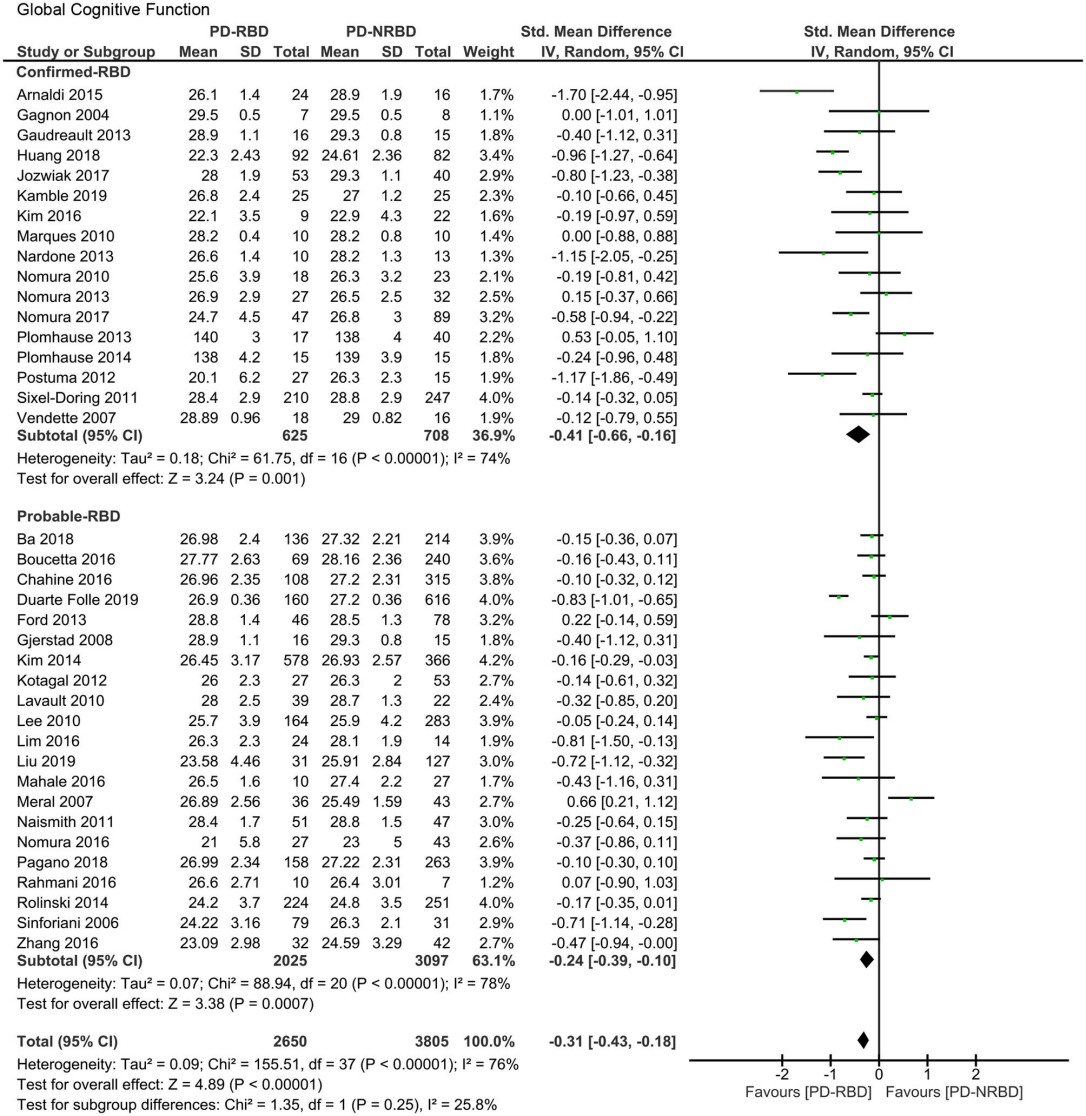
Forest plot for global cognitive function with subtotals by the diagnosis of rapid eye movement sleep behavior disorder (RBD) displaying the effect size calculated using a random-effects model. *SD*, standard deviation; *Std. Mean Difference*, standardized mean difference; *CI*, confidence interval.

The meta-analysis included 17 “Confirmed-RBD” and 21 “Probable-RBD” studies. For the “Confirmed-RBD” subgroup, PD-RBD patients had significantly lower scores than PD-NRBD patients, with a medium ES (SMD = −0.41, 95% CI = −0.66 to −0.16, *P* = 0.001); heterogeneity was moderate (*I*^2^ = 74%). For the “Probable-RBD” subgroup, PD-RBD patients also had significantly lower scores than did PD-NRBD patients, with a medium ES (SMD = −0.24, 95% CI = −0.39 to −0.10, *P* = 0.0007); heterogeneity was high (*I*^2^ = 78%). No significant difference between these two subgroups was observed (Table 3, Figure 2).

**Table 3:** Summary of the meta-analytic results of the following cognitive dimensions.

**Domains/subgroups**	***K***	**PD-RBD**	**PD-NRBD**	**Effect size**		**Heterogeneity**	
				**SMD (*P*-value)**	**95% CI**	***I*^**2**^ (%)**	***P***
**CONFIRMED-RBD**							
Global cognitive function	17	625	708	−0.41 (**0.001**)	−0.66 to −0.16	74	<0.00001
Memory—long-term verbal recall	5	128	109	−0.64 (**0.02**)	−1.16 to −0.11	70	0.009
Memory—long-term verbal recognition	3	93	74	−0.50 (**0.002**)	−0.81 to −0.19	0	0.58
Memory—short-term verbal recall	2	35	35	0.15 (0.63)	−0.47 to 0.78	36	0.21
EF—generativity	5	128	109	−1.12 (**0.002**)	−1.85 to −0.39	83	0.0001
EF—inhibition	5	128	99	0.63 (**0.0007**)	0.27–1.00	36	0.18
EF—shifting	3	93	74	0.80 (**<0.00001**)	0.48–1.12	0	0.66
EF—updating	4	110	93	−0.39 (0.22)	−1.01 to 0.23	75	0.007
Language	2	68	55	−0.49 (**0.009**)	−0.85 to −0.12	0	0.93
Visuospatial/constructional ability	3	93	74	−0.61 (**0.0001**)	−0.92 to −0.30	0	0.99
**PROBABLE-RBD**							
Global cognitive function	21	2025	3097	−0.24 (**0.0007**)	−0.39 to −0.10	78	<0.00001
Memory—long-term verbal recall	6	476	714	−0.20 (0.18)	−0.49 to 0.09	81	<0.0001
Memory—long-term verbal recognition	5	470	877	0.07 (0.64)	−0.21 to 0.34	80	0.0004
Memory—short-term verbal recall	2	111	73	−0.23 (0.24)	−0.61 to 0.16	35	0.22
EF—generativity	7	763	1368	0.12 (0.49)	−0.22 to 0.45	92	<0.00001
EF—inhibition	2	68	89	0.40 (0.27)	−0.31 to 1.11	79	0.03
EF—shifting	4	198	163	0.39 (**0.0003**)	0.18–0.61	0	0.68
EF—updating	2	83	89	−0.34 (0.11)	−0.76 to 0.08	46	0.17
Visuospatial/constructional ability	6	539	1117	−0.04 (0.78)	−0.37 to 0.28	88	<0.00001
**MIXED-RBD**							
Memory—long-term visual recall	4	172	172	−0.34 (**0.002**)	−0.55 to −0.12	0	0.70
Memory—short-term spatial recall	2	104	56	−0.65 (**0.001**)	−1.04 to −0.26	19	0.27
EF—general	4	721	444	−0.31 (**0.02**)	−0.57 to −0.06	46	0.13
Processing speed/complex attention/working memory (LNS)	4	412	802	−0.01 (0.95)	−0.33 to 0.30	81	0.001
Processing speed/complex attention/working memory (TMT: A)	3	103	98	0.57 (**<0.0001**)	0.29–0.86	0	0.82
Psychomotor ability	6	464	1084	−0.31 (0.05)	−0.62 to 0.01	84	<0.00001

*Statistically significant values of effect size are reported in bold*.

*K, number of studies; PD-RBD, number of PD patients with RBD; PD-NRBD, number of PD patients without RBD; SMD, standardized mean difference; CI, confidence intervals; I^2^, heterogeneity statistics; EF, executive function; LNS, letter–number sequencing; TMT: A, trail making test: part A*.

Global cognitive function is the only domain where the second subgroup analysis could be performed. The meta-analysis included six “Mediated by Clonazepam” studies and three “Unmediated by Clonazepam” studies. For the “Mediated by Clonazepam” subgroup, PD-RBD patients had significantly lower scores than did PD-NRBD patients, with a medium ES (SMD = −0.31, 95% CI = −0.51 to −0.12, *P* = 0.001); heterogeneity was moderate (*I*^2^ = 56%). For the “Unmediated by Clonazepam” group, the ES was not significant. No significant difference between these two subgroups was observed (Supplementary Figure 1).

#### Memory—Long-Term Verbal Recall

**Figure 3:**
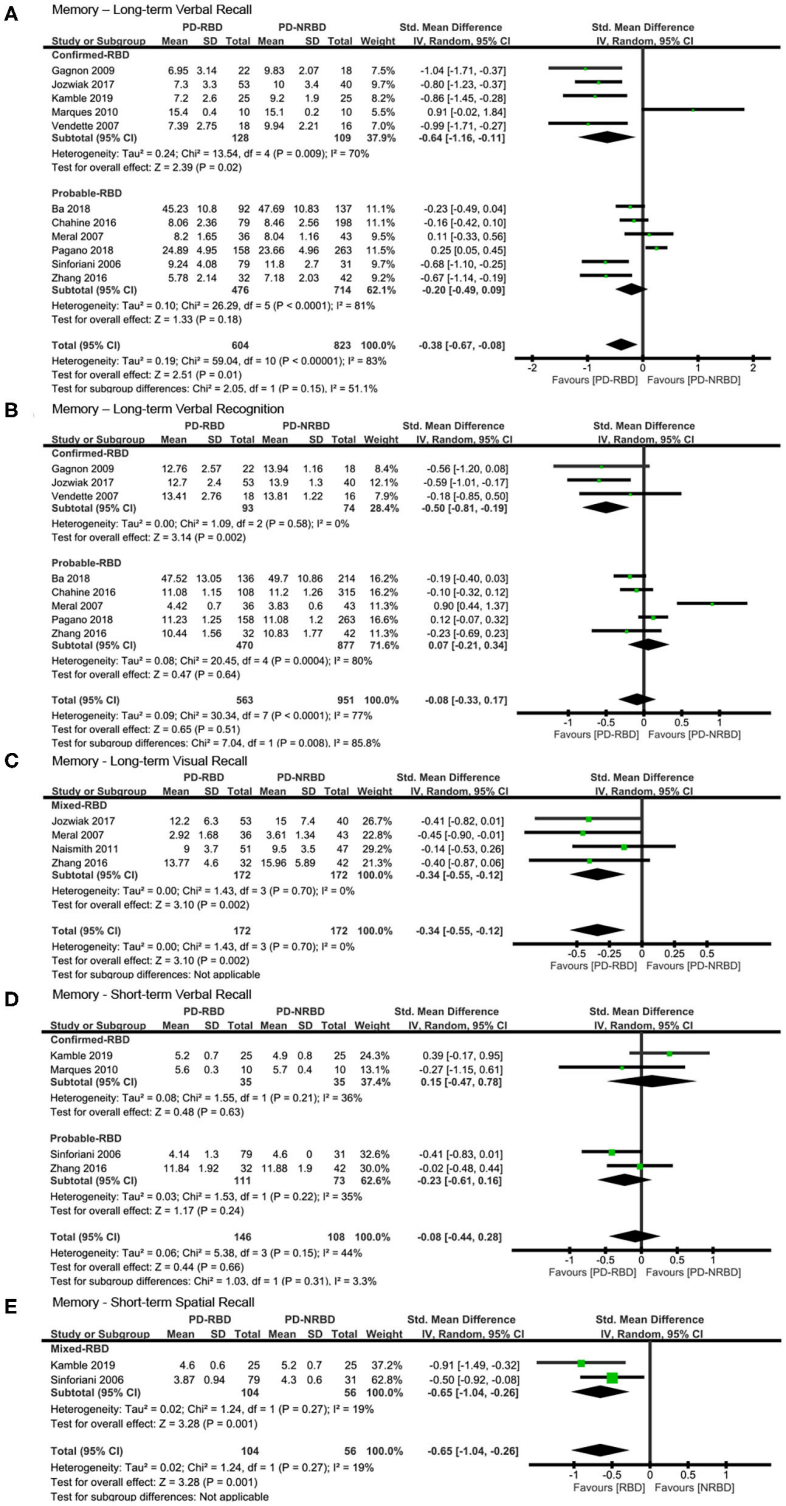
Forest plot for **(A)** long-term verbal recall with subtotals by the diagnosis of rapid eye movement sleep behavior disorder (RBD), **(B)** long-term verbal recognition with subtotals by the diagnosis of RBD, **(C)** long-term visual recall, **(D)** short-term verbal recall with subtotals by the diagnosis of RBD, and **(E)** short-term spatial recall displaying effect size calculated using a random-effects model. *SD*, standard deviation; *Std. Mean Difference*, standardized mean difference; *CI*, confidence interval.

The meta-analysis included five “Confirmed-RBD” and six “Probable-RBD” studies. For the “Confirmed-RBD” subgroup, PD-RBD patients had significantly lower scores than did PD-NRBD patients, with a medium ES (SMD = −0.64, 95% CI = −1.16 to −0.11, *P* = 0.02); heterogeneity was moderate (*I*^2^ = 70%). For the “Probable-RBD” subgroup, the ES was not significant. No significant difference between these two subgroups was observed (Table 3, Figure 3A).

#### Memory—Long-Term Verbal Recognition

The meta-analysis included three “Confirmed-RBD” and five “Probable-RBD” studies. For the “Confirmed-RBD” subgroup, PD-RBD patients had significantly lower scores than did PD-NRBD patients, with a medium ES (SMD = −0.50, 95% CI = −0.81 to −0.19, *P* = 0.002); heterogeneity was absent (*I*^2^ = 0%). For the “Probable-RBD” subgroup, the ES was not significant. The difference between these two subgroups was significant (*P* = 0.008) (Table 3, Figure 3B).

#### Memory—Long-Term Visual Recall

The meta-analysis included one “Confirmed-RBD” and three “Probable-RBD” primary studies. Given the exiguity of the primary studies in the “Confirmed-RBD” subgroup, we analyzed these two subgroups together. PD-RBD patients had significantly lower scores than did PD-NRBD patients, with a medium ES (SMD = −0.34, 95% CI = −0.55 to −0.12, *P* = 0.002); heterogeneity was absent (*I*^2^ = 0%) (Table 3, Figure 3C).

#### Memory—Short-Term Verbal Recall

This meta-analysis included two “Confirmed-RBD” and two “Probable-RBD” studies. The ESs for both groups were insignificant (Table 3, Figure 3D).

#### Memory—Short-Term Spatial Recall

This meta-analysis included one “Confirmed-RBD” and one “Probable-RBD” study. Given the exiguity of the primary studies in both subgroups, we analyzed them together. PD-RBD patients had significantly lower scores than did PD-NRBD patients, with a medium ES (SMD = −0.65, 95% CI = −1.04 to −0.26, *P* = 0.001); heterogeneity was absent (*I*^2^ = 19%) (Table 3, Figure 3E).

#### General EF

**Figure 4:**
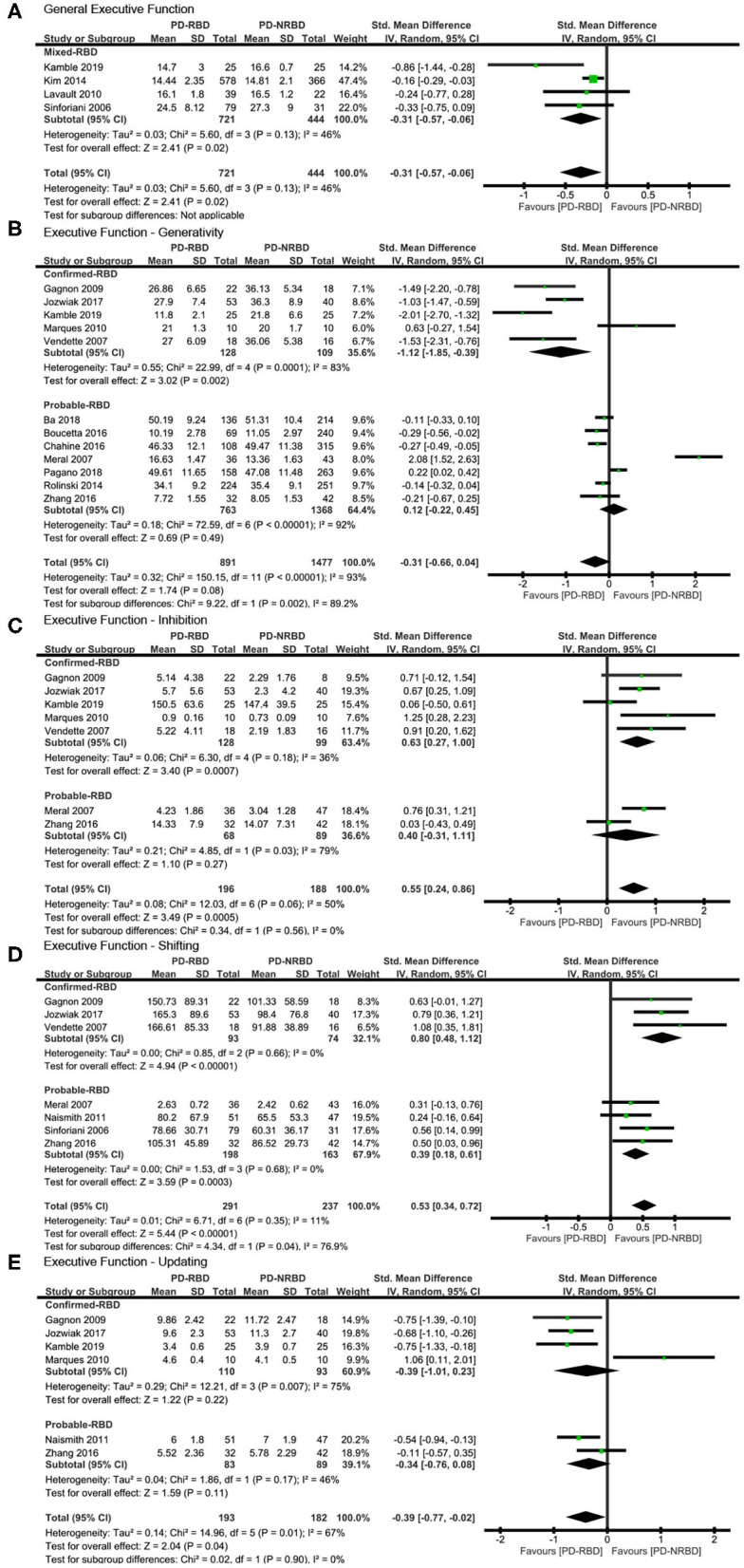
Forest plot for **(A)** general executive function (EF), **(B)** generativity with subtotals by the diagnosis of rapid eye movement sleep behavior disorder (RBD), **(C)** inhibition with subtotals by the diagnosis of RBD, **(D)** shifting with subtotals by the diagnosis of RBD, and **(E)** updating with subtotals by the diagnosis of RBD displaying effect size calculated using a random-effects model. *SD*, standard deviation; *Std. Mean Difference*, standardized mean difference; *CI*, confidence interval.

The meta-analysis included one “Confirmed-RBD” and three “Probable-RBD” studies. Given the exiguity of the primary studies in the “Confirmed-RBD” subgroup, we analyzed these two subgroups together. PD-RBD patients had significantly lower scores than did PD-NRBD patients, with a medium ES (SMD = −0.31, 95% CI = −0.57 to −0.06, *P* = 0.02); heterogeneity was low (*I*^2^ = 46%) (Table 3, Figure 4A).

#### EF—Fluid Reasoning

The meta-analytic study could not be performed due to the exiguity of the primary studies (*n* = 1). PD-RBD patients performed worse in this domain than did PD-RBD patients, according to the only primary study.

#### EF—Generativity

The meta-analysis included five “Confirmed-RBD” and seven “Probable-RBD” studies. For the “Confirmed-RBD” subgroup, PD-RBD patients had significantly lower scores than did PD-NRBD patients, with a large ES (SMD = −1.12, 95% CI = −1.85 to −0.39, *P* = 0.002); heterogeneity was high (*I*^2^ = 83%). For the “Probable-RBD” subgroup, the ES was not significant. The difference between these two subgroups was significant (*P* = 0.002) (Table 3, Figure 4B).

#### EF—Inhibition

The meta-analysis included five “Confirmed-RBD” and two “Probable-RBD” studies. For the “Confirmed-RBD” subgroup, PD-RBD patients had significantly higher scores than did PD-NRBD patients, with a medium ES (SMD = 0.63, 95% CI = 0.27–1.00, *P* = 0.0007); heterogeneity was low (*I*^2^ = 36%). For the “Probable-RBD” subgroup, the ES was not significant. No significant difference between these two subgroups was observed (Table 3, Figure 4C).

#### EF—Shifting

The meta-analysis included three “Confirmed-RBD” studies and four “Probable-RBD” primary studies. For the “Confirmed-RBD” subgroup, PD-RBD patients had significantly higher scores than did PD-NRBD patients, with a large ES (SMD = 0.80, 95% CI = 0.48–1.12, *P* < 0.00001); heterogeneity was absent (*I*^2^ = 0%). For the “Probable-RBD” subgroup, PD-RBD patients also had significantly higher scores than did PD-NRBD patients, with a medium ES (SMD = 0.39, 95% CI = 0.18–0.61, *P* = 0.0003); heterogeneity was absent (*I*^2^ = 0%). The difference between these two subgroups was significant (*P* = 0.04) (Table 3, Figure 4D).

#### EF—Updating

This meta-analysis included four “Confirmed-RBD” and two “Probable-RBD” studies. The ESs for both subgroups were insignificant (Table 3, Figure 4E).

#### Language

**Figure 5:**
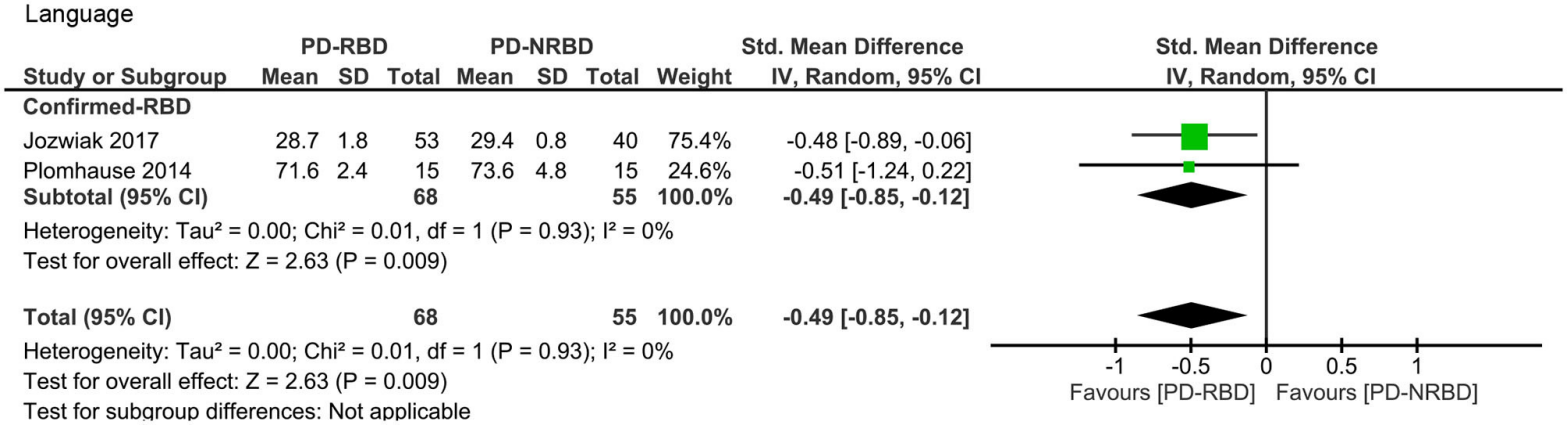
Forest plot for language with subtotals by the diagnosis of rapid eye movement sleep behavior disorder (RBD) displaying effect size calculated using a random-effects model. *SD*, standard deviation; *Std. Mean Difference*, standardized mean difference; *CI*, confidence interval.

This meta-analysis only included two “Confirmed-RBD” studies. PD-RBD patients had significantly lower scores than did PD-NRBD patients, with a medium ES (SMD = −0.49, 95% CI = −0.85 to −0.12, *P* = 0.009); heterogeneity was absent (*I*^2^ = 0%) (Table 3, Figure 5).

#### Processing Speed/Complex Attention/Working Memory

This domain was evaluated by seven studies in total, four of which used the letter–number sequence (LNS) and three adopted the trail making test, part A (TMT: A). Since these two tests could not be combined together, we analyzed them separately.

**Figure 6:**
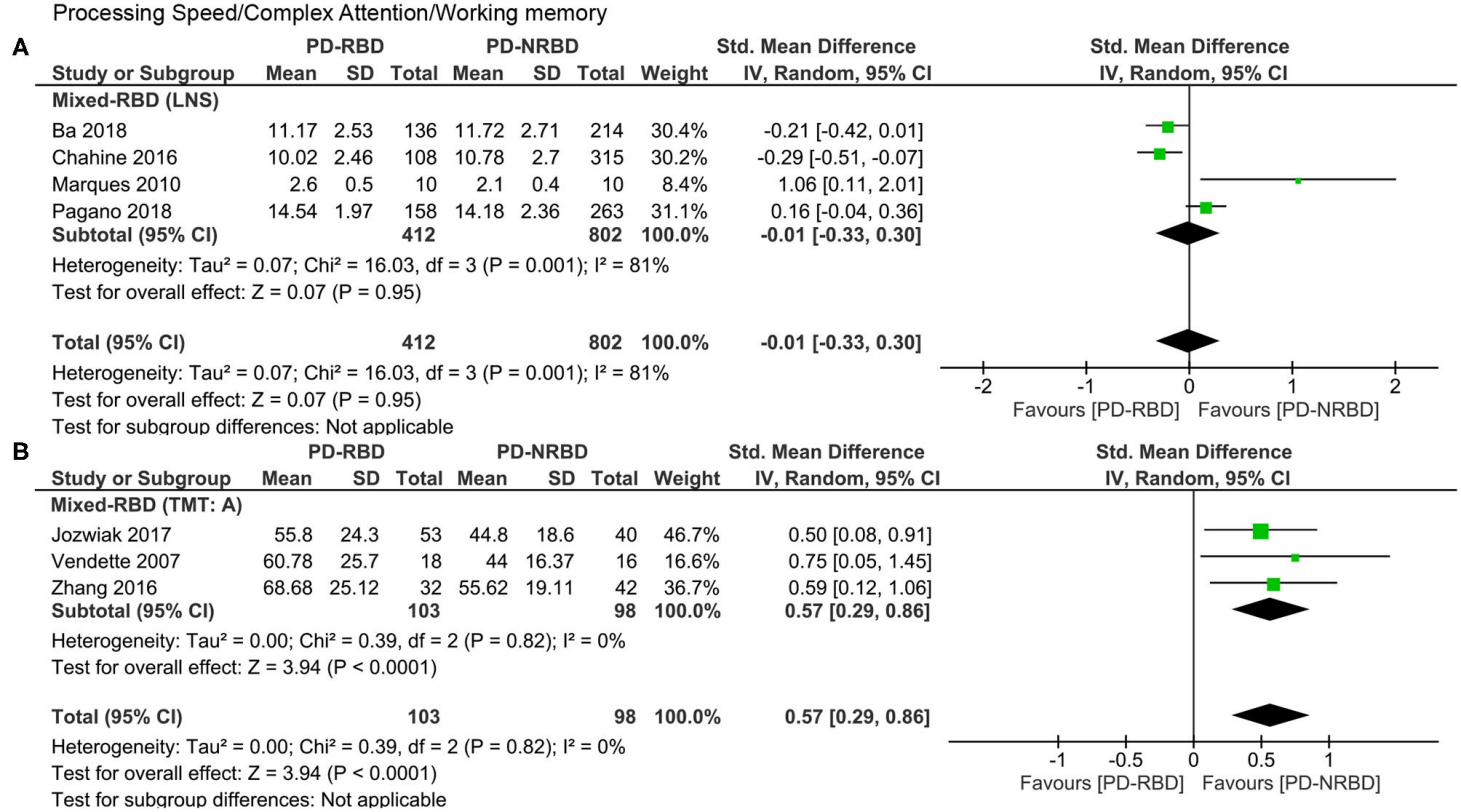
Forest plot for processing speed/complex attention/working memory evaluated by the **(A)** LNS and the **(B)** TMT: A displaying effect size calculated using a random-effects model. *SD*, standard deviation; *Std. Mean Difference*, standardized mean difference; *CI*, confidence interval; *LNS*, letter–number sequence; *TMT: A*, trail making test, part A.

The meta-analysis focusing only on studies that used LNS scores included one “Confirmed-RBD” and three “Probable-RBD” primary studies. Given the exiguity of the primary studies in the “Confirmed-RBD” subgroup, we analyzed these two subgroups together. The ES was not significant (Table 3, Figure 6A).

The meta-analysis focusing only on studies that used TMT: A scores included two “Confirmed-RBD” and one “Probable-RBD” primary studies. Given the exiguity of the primary studies in the “Probable-RBD” subgroup, we analyzed these two subgroups together. PD-RBD patients had significantly higher scores than did PD-NRBD patients, with a medium ES (SMD = 0.57, 95% CI = 0.29–0.86, *P* < 0.0001); heterogeneity was absent (*I*^2^ = 0%) (Table 3, Figure 6B).

#### Visuospatial and Constructional Ability

**Figure 7:**
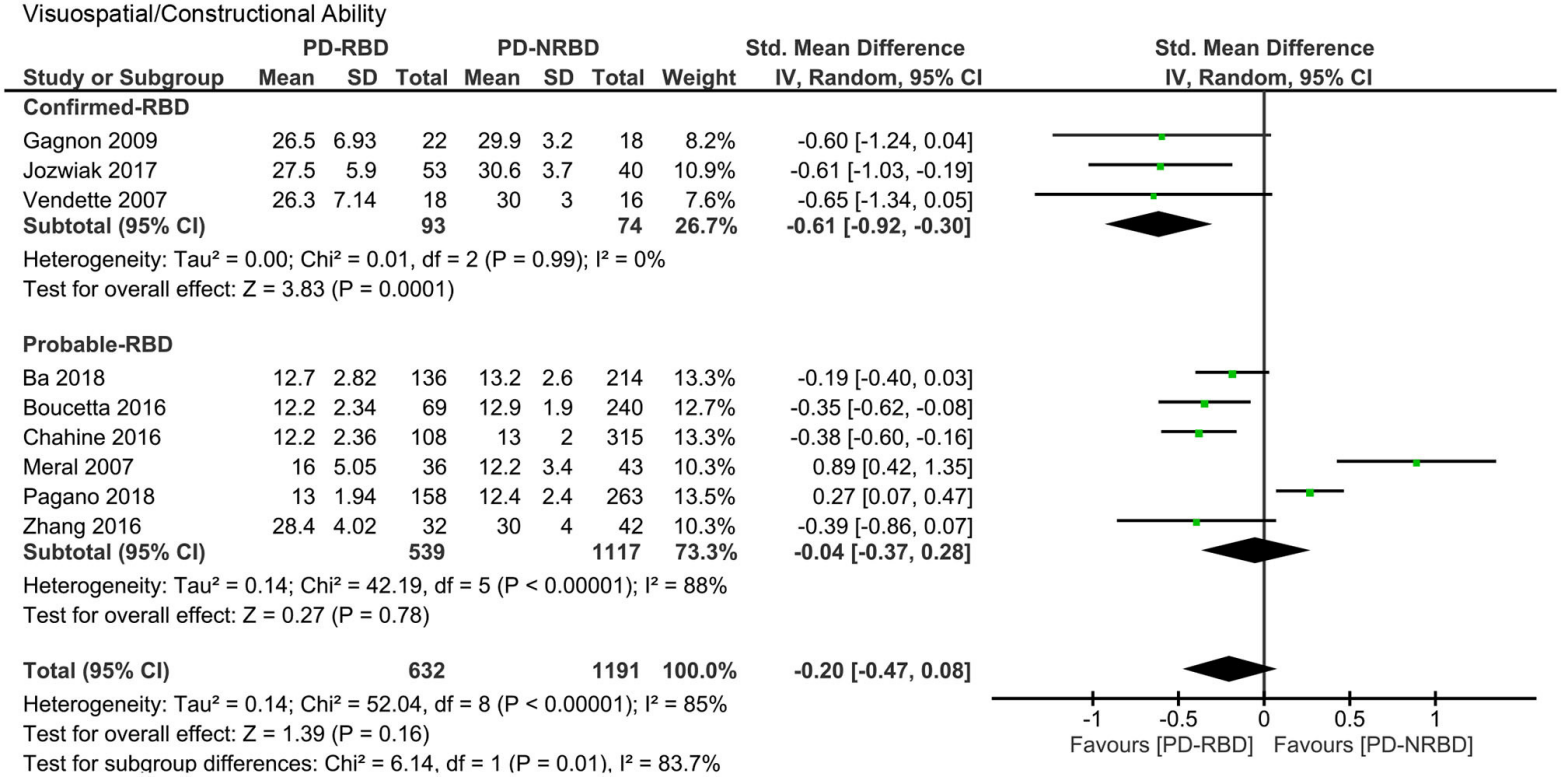
Forest plot for visuospatial/constructional ability with subtotals by the diagnosis of rapid eye movement sleep behavior disorder (RBD) displaying effect size calculated using a random-effects model. *SD*, standard deviation; *Std. Mean Difference*, standardized mean difference; *CI*, confidence interval.

The meta-analysis included three “Confirmed-RBD” and six “Probable-RBD” studies. For the “Confirmed-RBD” subgroup, PD-RBD patients had significantly lower scores than did PD-NRBD patients, with a medium ES (SMD = −0.61, 95% CI = −0.92 to −0.30, *P* = 0.0001); heterogeneity was absent (*I*^2^ = 0%). For the “Probable-RBD” subgroup, the ES was not significant. The difference between these two subgroups was significant (*P* = 0.01) (Table 3, Figure 7).

#### Psychomotor Ability

**Figure 8:**
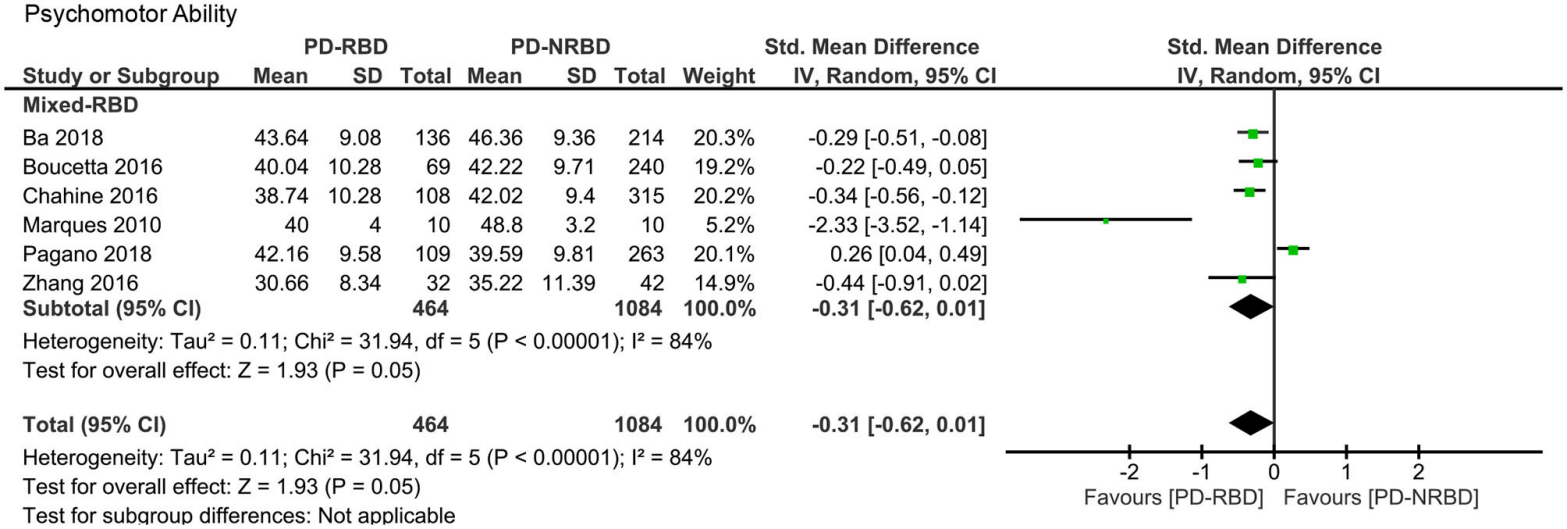
Forest plot for psychomotor ability displaying effect size calculated using a random-effects model. *SD*, standard deviation; *Std. Mean Difference*, standardized mean difference; *CI*, confidence interval.

The meta-analysis included one “Confirmed-RBD” and five “Probable-RBD” primary studies. Given the exiguity of the primary studies in the “Confirmed-RBD” subgroup, we analyzed these two subgroups together. The ES was not significant (Table 3, Figure 8).

### Moderator Analysis

Meta-regression revealed that gender had a significant impact on the obtained ES for psychomotor ability (*K* = 6, β = 6.310, *P* = 0.001); PD duration for psychomotor ability (*K* = 6, β = −0.225, *P* = 0.005); H&Y for visuospatial/constructional ability (*K* = 6, β = −0.835, *P* = 0.033); LEDD for psychomotor ability (*K* = 6, β = −0.003, *P* = 0.005); cognitive test for global cognitive function (*K* = 38, β = 0.221, *P* = 0.041), long-term verbal recall (*K* = 11, β = 0.135, *P* = 0.039), long-term verbal recognition (*K* = 8, β = 0.336, *P* = 0.000), inhibition (*K* = 7, β = 0.558, *P* = 0.011), and visuospatial/constructional ability (*K* = 9, β = −0.576, *P* = 0.048); and RBD assessment for generativity (*K* = 12, β = 1.294, *P* = 0.013), shifting (*K* = 7, β = −0.417, *P* = 0.033), and psychomotor ability (*K* = 6, β = 2.239, *P* = 0.001). No other demographic and clinical factors manifested any significant effect on the ES for these outcomes. Finally, no other aspects had significant impact on the ES for the remaining outcomes (Supplementary Table 4).

### Sensitivity Analysis

No obvious outliers were uncovered by the sensitivity analyses aiming at determining the effect of any individual study on the pooled ES, indicating the stability of the meta-analytic findings (Supplementary Figures 2–9).

Another sensitivity analysis identified that the study by Marques et al. (66) contributed dramatically to the heterogeneities in the “Confirmed-RBD” subgroups in the long-term verbal recall and updating domains, separately. After excluding this study, all heterogeneities plunged to 0%, and SMD decreased from −0.64 (95% CI = −1.16 to −0.11, *P* = 0.02) to −0.89 (95% CI = −1.17 to −0.61, *P* < 0.00001) in long-term verbal recall and from −0.39 (95% CI = −1.01 to 0.23, *P* = 0.22) to −0.71 (95% CI = −1.02 to −0.41, *P* < 0.00001) in updating. For the rest of the domains, excluding one single study did not change the heterogeneity dramatically.

### Publication Bias

Global cognitive domain was the only domain where publication bias analysis could be performed, and the funnel plot for it suggested symmetry: Egger's test was insignificant and the trim-and-fill analysis did not remove any study for both the Confirmed and Probable-RBD subgroups.

## Discussion

### Summary of Findings

This study is the first systematic review and meta-analysis of the association between RBD and cognitive dysfunctions in patients with PD. This meta-analysis indicates that, relative to those without RBD, people with PD who were diagnosed with RBD, as confirmed or probable, demonstrate poorer cognitive performance that differs across cognitive domains. Specifically, Confirmed-RBD patients performed more poorly than those without RBD in global cognitive function, long-term verbal recall, long-term verbal recognition, generativity, inhibition, shifting, language, and visuospatial/constructional ability; Probable-RBD, in global cognitive function and shifting; and Mixed-RBD, in long-term visual recall, short-term spatial recall, general EF, and processing speed/complex attention/working memory that was evaluated by the TMT: A.

Our results put emphasis on PSG that provides objective statistics with which to compare subjective accounts in diagnosing RBD. Regarding the range and degree of cognitive damage, we found that Confirmed-RBD patients were more serious than did Probable-RBD patients when both were compared to PD-NRBD patients. Dream enactment behavior, the major diagnostic basis of Probable-RBD, is not specific for RBD (1, 94, 95), and presumably, Probable-RBD patients diagnosed accordingly are less generalizable to RBD patients. The results from our analyses clearly present the difference between patients with PSG-confirmed RBD and those with probable RBD based on subjective complaints. Although the gold standard for assessing RBD remains the laboratory PSG, there is heightened growing interest in home-based sleep monitoring by portable or wearable monitoring devices (96, 97). Identification of confirmed RBD cases will likely grow with advances in technology enabling home-based PSG assessment.

Clonazepam, the drug of choice in the treatment of RBD, was reported to deteriorate cognition, as noted above. Although PD-RBD patients treated with clonazepam performed significantly worse on global cognitive function compared to PD-NRBD patients while the unmediated group did not manifest any difference, the subgroup difference was statistically insignificant. This seeming contradiction needs to be further explored by large-sized investigations.

One sensitivity analysis revealed no outlier, and another identified that the results reported by Marques et al. (66) and Pagano et al. (79) contributed significantly to the heterogeneities found in the analysis of long-term verbal recall and updating. The investigations showed that the results of the tests were conflicting with the other studies in their respective domains and were statistically insignificant, which means that the PD-RBD patients performed insignificantly better than did PD-NRBD patients. These two primary studies did not mention the reason for these dissimilar results.

Some demographics and clinical phenomenology of RBD (30) and PD-RBD (25, 73) were identified, such as male gender, age at onset, and severity of PD. Therefore, these features could also affect the relationship between RBD and cognitive dysfunctions. Moderator analysis supported our hypothesis that the neuropsychological patterns of PD-RBD patients are dependent on some demographic and clinical aspects. Moreover, it also revealed the effects of evaluating cognition or RBD on some cognitive domains like global cognitive function. However, considering that the clinical data of patients were not reported consistently across studies, these results should be interpreted with more caution and examined further.

Many studies have revealed no relationship between sleep-related deficits and cognition when insensitive neuropsychological tests were used. For instance, the Mini-Mental State Examination (MMSE), designed to detect frank dementia (98) instead of cognitive dysfunction in PD, possesses a “strikingly low sensitivity” at only 50% when used to screen for dementia in people with PD (99). MMSE is thought to be less sensitive than the Montreal Cognitive Assessment (MOCA) in detecting MCI in PD patients (100). Although the MMSE was not recommended to evaluate cognition in PD by the 2010 Movement Disorders Task Force to detect cognitive impairment in PD (98), it remained the most commonly used global cognitive test. Impaired global cognitive functions detected mostly by the MMSE were related to “bad sleep” or “pure apathy” (51, 53) and were insignificantly associated with impulse control disorders (52) in PD patients in previous meta-analyses. In our meta-analysis, the MMSE was used in 22 of the 38 included studies assessing global cognition. Similarly, the Frontal Assessment Battery (FAB), designed to recognize frontal lobe dysfunction, has been validated in frontotemporal dementia, progressive supranuclear palsy (PSP), and PD. Regression analysis proved that 69.7% of individuals with PSP and frontotemporal dementia were classified correctly with FAB, which suggested that deficits associated with predominantly medial–prefrontal dysfunction could be captured successfully by FAB (101). However, FAB is insensitive to cognitive damage in PD because it detects frontal lobe dysfunction instead of disorders that primarily involve the dorsolateral and ventrolateral prefrontal cortices in PD (102). A study identified the sensitivity (66.3%) and specificity (72.3%) of FAB in detecting dementia in PD at a cutoff of 26 points (103). All the enrolled studies evaluating general EF used FAB in our meta-analysis. Thus, these two domains need to be confirmed by more sensitive tests. Moreover, due to the conflicting results of the processing speed/complex attention/working memory domain evaluated with LNS and TMT: A, this domain also need to be confirmed further.

### The Process of Selecting Neurophysiological Tests

In diagnosing MCI in PD, Litvan et al. (48) suggested that two highly similar tests (e.g., two list learning tests or two story recall tests) or highly correlated scores from the same test (e.g., immediate and delayed recall of a word list) should not be used to meet the MCI criterion for two test-score abnormalities. As mentioned earlier, when a cognitive domain was determined with more than one test in a study, we extracted and analyzed data from the most sensitive, relevant, and frequently used tests and discussed in detail the process of assigning the tests in these domains.

In the global cognitive function domain, among the enrolled primary studies, eight (33, 60, 61, 69, 71–74) employed both the MMSE and MOCA. Because the sensitivity of the MOCA is higher than that of the MMSE in evaluating cognitive decline in PD (98–100), we analyzed the MOCA results of these eight studies.

In the long-term verbal recall domain, the tests used were several highly similar tests. We extracted the results of the delayed recall tasks from these tests, like Litvan et al. (48) who suggested in diagnosing MCI in PD or Jansen et al. (104) who selected evaluating cognition in individuals with MCI. This criterion was also applicable to the long-term visual recall domain.

Six studies (5, 31, 33, 35, 66, 82) used both verbal fluency, semantic and letter, to evaluate the generativity. It is controversial which aspect of verbal fluency was more affected in PD patients (105–107). In an attempt to resolve the inconsistency, a meta-analysis with 2,644 PD patients showed that, although PD patients manifested greater deficits in semantic than in letter fluency, the difference was small (108). Because several other studies only employed semantic verbal fluency, we extracted semantic results from these six studies and combined them with the results from other studies in order to minimize heterogeneity. Kamble et al. (61) used two highly similar tests in this domain, the semantic verbal fluency and animal naming tests. Similarly, we extracted the results from the semantic fluency test. This criterion was also applicable to the shifting domain where two tasks of the TMT were adopted in a study (5); we analyzed the task employed by other studies as well. Moreover, two tests, Stroop task and TMT, can be measure by both speed and accuracy; we extracted speed data when these are available.

Concerning the visuospatial and constructional ability, six different tests were adopted by the enrolled primary studies. The Benton judgment of line orientation test (BJLOT), one of the most extensively used visuospatial tasks, was sensitive to visuospatial deficits in PD (109). The copy of the Rey–Osterrieth complex figure test is another widely used test to assess visuo-constructional ability. However, due to its complexity, EF is also reflected in this test (110–112). Using the clock drawing test, PD patients manifested a low performance compared with healthy controls (113). However, the major reason for clock drawing difficulties in PD with early cognitive impairment is dysfunctional executive control of memory retrieval instead of visuospatial impairment (114). Similarly, the block design measures visual perception and organization and visual–motor coordination, and also non-verbal reasoning, analysis, and synthesis (115). The bells test is used to investigate visual perception, processing speed, and attention (116). However, it is considered a sensitive test to diagnose visual hemineglect (117, 118). The sensitivity of Benton's facial recognition in PD patients is rarely studied. A study pointed out that medicated people with PD did not show significant deficits in this test compared with those untreated (119). Therefore, in this domain, the BJLOT is our priority when it exists with other tests of this domain in one study, and the copy of the Rey–Osterrieth complex figure test is our second choice.

### Possible Mechanisms of “RBD-PD Phenotype”

Although the relationship between RBD and cognitive dysfunction in PD was confirmed based on our results, the mechanisms behind this phenomenon are yet to be elucidated. The following dysregulations that affect both RBD and cognitive dysfunction in PD may be the targets.

Cholinergic dysfunction is strongly associated with RBD and cognitive decline in PD. RBD in the context of α-synucleinopathies was suggested to be a result of degeneration of the pontomedullary cholinergic pathways (67, 120, 121). A smaller volume of the pontomesencephalic tegmentum was found in PD patients with RBD than in those without (76). Moreover, dysfunctions of cholinergic systems and their projections were consistently associated with cognitive damage in PD (122–127). Cholinergic pedunculopontine nucleus neuronal loss in PD is believed to be attributable to cognitive damage (128–130). The decreased volume and the disrupted resting-state functional connectivity of the basal nucleus of Meynert (BNM), the main source of cholinergic innervation (131, 132), were found to be correlated with cognitive decline in PD (133–135). Rivastigmine, an inhibitor of acetylcholinesterase and butyrylcholinesterase, is effective in treating RBD and dementia associated with PD separately (136, 137).

Nigro-striatal dopaminergic impairment, limbic dysfunction, inflammation, and altered metabolism are also related to RBD and cognitive damage in PD. In PD-RBD patients, more severe nigro-striatal dopaminergic damage (138) and greater dopamine transporter loss (139) were discovered compared with PD-NRBD patients. A positive relationship between striatal dopamine transporter availability and fundamental cognitive capability was determined in PD patients (140). Compared to PD-NRBD patients, smaller volumes of the hypothalamus, thalamus, amygdala, anterior cingulate cortex, left posterior cingulate, and hippocampus were found in PD-RBD patients (64, 76). The volume loss of the thalamus and the accompanying damaged functional connectivity were also observed in PD patients with MCI (141). Elevations of peripheral inflammatory factors were found in the PD-RBD group compared with the PD-NRBD group (142). Cognitive damage in PD patients was associated with a higher level of circulating lymphocytes and—in drug-naive ones at least—with dysregulation of the T regulatory cells (143). In addition, an altered brain glucose metabolism was observed in PD patients with RBD and MCI (138, 144–147).

Therefore, it was previously suggested that PD-RBD represents a unique subtype of PD with severe non-motor symptoms. The positive relationship between RBD and cognitive decline in PD patients according to our results enriched and expanded this opinion. The above-mentioned dysfunctions in PD patients accompanied with either RBD or cognitive decline elucidated this relationship further, thus supplying possible therapeutic targets. Even though the results are encouraging, more cases and experiments are needed to confirm this phenotype.

### Strengths and Limitations

This meta-analysis not only confirms the relationship between RBD and cognitive dysfunction in PD but also specifies which cognitive domains are involved. In addition, this meta-analysis scientifically distinguished probable RBD from true RBD and thus demonstrated the difference between objective and subjective evaluation of RBD.

However, two limitations warrant consideration when interpreting our results and designing further studies. The first is the pooling of other non-motor symptoms such as hallucinations and depression, which could also affect the cognitive status of patients with PD. Several longitudinal reports have revealed that hallucination can be a predictor of cognitive dysfunction in PD (148–151), specifically in the domain of EF (152). Moreover, a study confirmed cross-sectionally and longitudinally that hallucination was significantly related to the presence and development of dementia (153), and another separately confirmed the relationships between hallucination and depression with dementia in PD (154). In addition, the relationship between depression and cognitive dysfunction in individuals with PD was also confirmed in several studies, and the consensus was that PD patients with baseline depression manifested deteriorated cognition and motor ability (155–157). Given that nearly 60% of PD patients manifested more than one non-motor symptom and roughly 25% displayed more than two (158), the relationship between “pure RBD” and cognitive decline in PD patients is difficult to detect. The second limitation is that the symptoms of cognitive damage and RBD can both fluctuate (28, 59, 159, 160), so studies evaluating them at an arbitrary time point may not comprehensively and accurately reflect the condition. In addition to the fluctuation of symptoms, the effect of the appearance order of RBD and PD symptoms on cognition is controversial (70, 161).

### Significance and Conclusion

This meta-analysis strongly suggests an association between RBD and cognitive dysfunctions in PD patients. Early and routine screening by cognitive tasks is simple and inexpensive and should be part of the standard assessment of all PD-RBD patients before they evolve to irreversible dementia in the study setting. Currently, there are no pharmacological therapeutics that could slow cognitive decline or dementia (162–164); however, there is some evidence suggesting that non-pharmacological interventions, like cognitive training, could enhance cognition in non-demented early-stage PD patients (165–167). This underscores the importance of a timely intervention of cognitive dysfunctions in PD. Our findings should be extended in larger prospective longitudinal studies to assess the progression of both cognitive decline and RBD in PD and to identify moderators that may help in a personalized care approach.

## Author Contributions

JM and JG developed the concept for the study. JM and JY carried out search, quality assessment, and initial data interpretation. JM, XH, and LC carried out statistical analysis. JM prepared the manuscript draft, with input and revisions from LC, YH, BT, and JG. All authors approved the final version.

## Conflict of Interest

The authors declare that the research was conducted in the absence of any commercial or financial relationships that could be construed as a potential conflict of interest.
