# Supplementary material for: Association Between REM Sleep Behavior Disorder and Cognitive Dysfunctions in Parkinson's Disease: A Systematic Review and Meta-Analysis of Observational Studies
Source: Front Neurol. 2020 Nov 6;11:577874. doi: 10.3389/fneur.2020.577874 (PMC7677514; doi:10.3389/fneur.2020.577874)
Supplement: Supplementary file 1 [file Data_Sheet_1.docx]

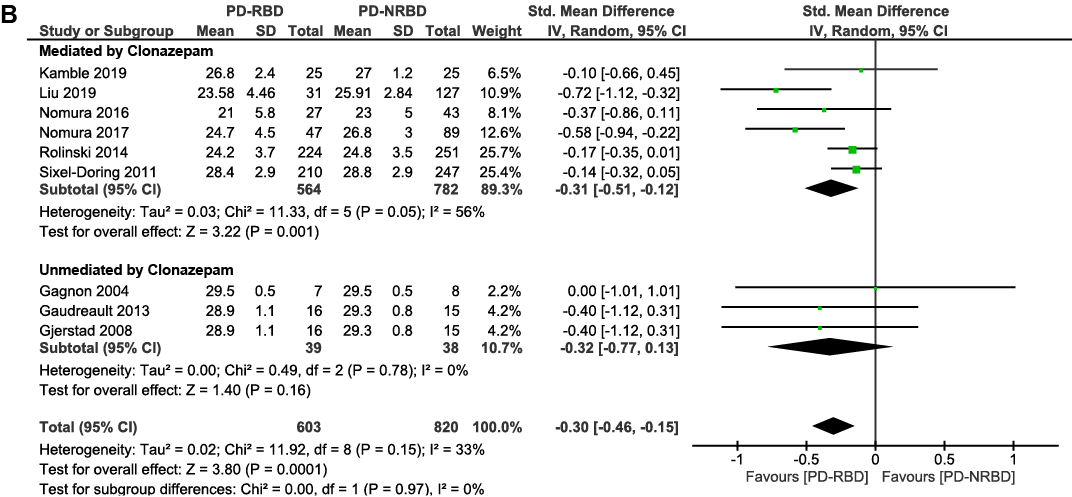


Figure S1. Forest plot for global cognitive function with subtotals by the medication of Clonazepam displaying effect size calculated using a random effects model. SD = standard deviation; Std. Mean Difference = Standardized mean difference; CI = Confidence interval.

**
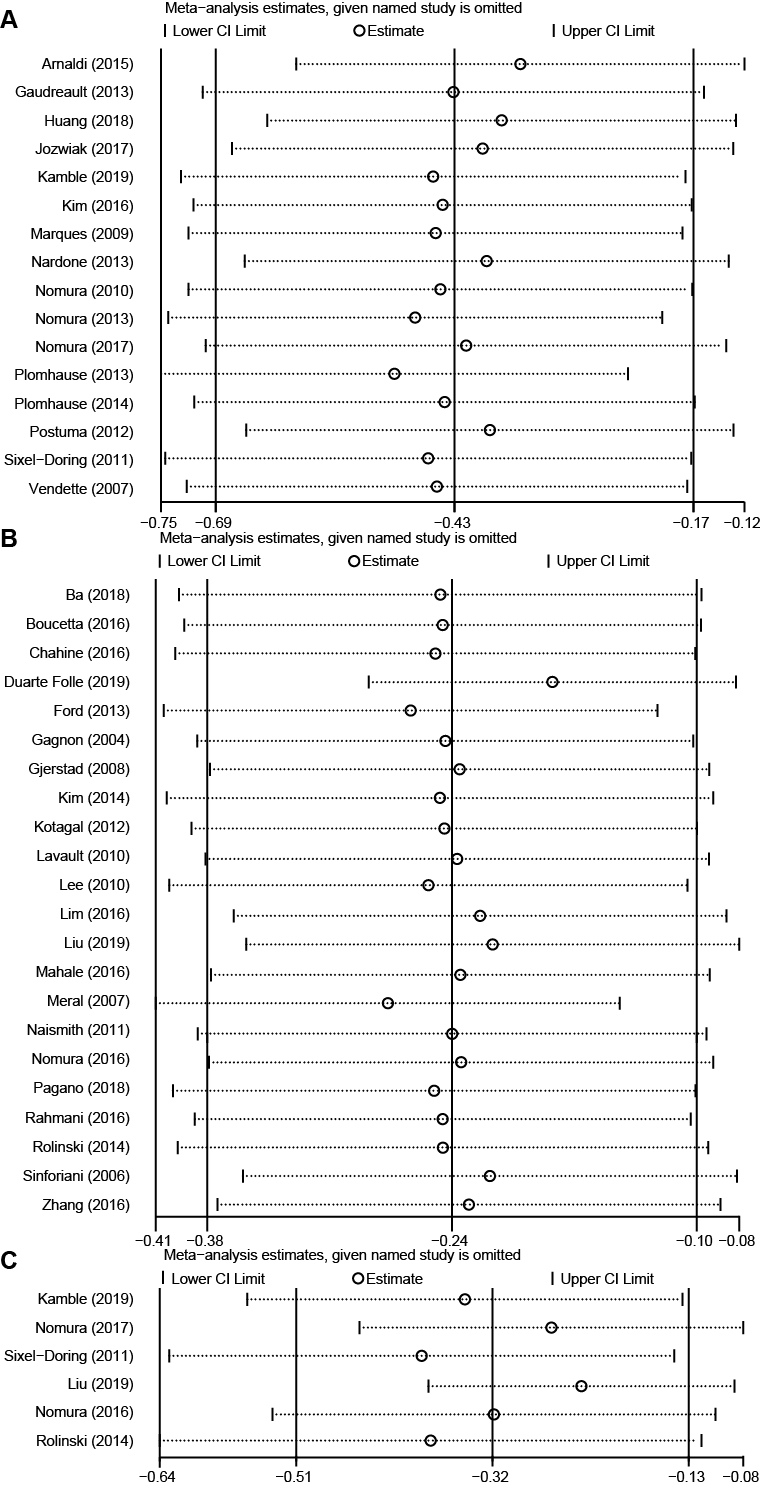
**

**Figure S2**. Sensitivity analysis of instability in (A) Confirmed-RBD subgroup, (B) Probable-RBD subgroup and (C) Mediated by Clonazepam subgroup in global cognitive function studies. CI, Confidence Intervals.


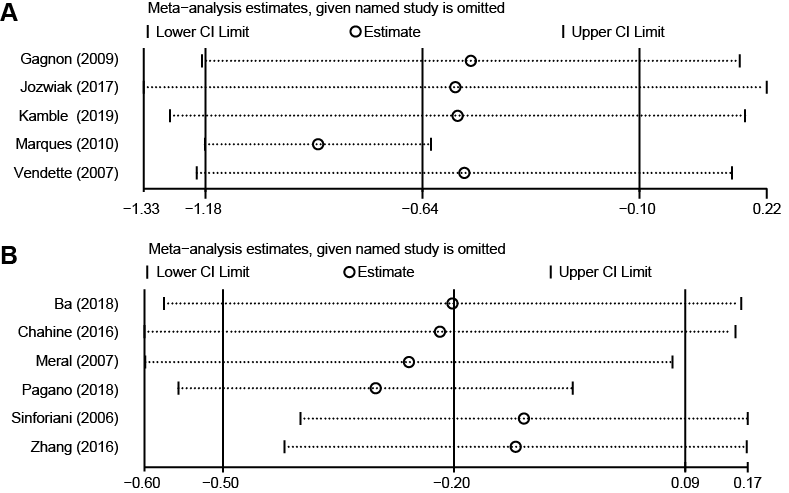


**Figure S3**. Sensitivity analysis of instability in (A) Confirmed-RBD subgroup and (B) Probable-RBD subgroup in long term verbal recall studies. CI, Confidence Intervals.


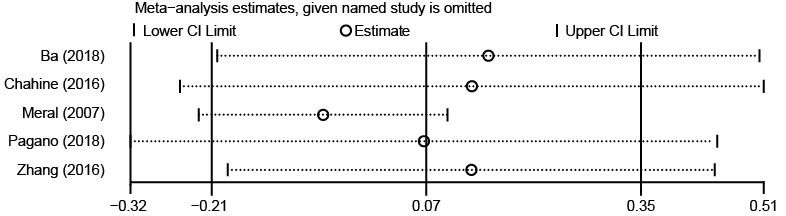


**Figure S4**. Sensitivity analysis of instability in Probable-RBD subgroup in long term verbal recognition studies. CI, Confidence Intervals.


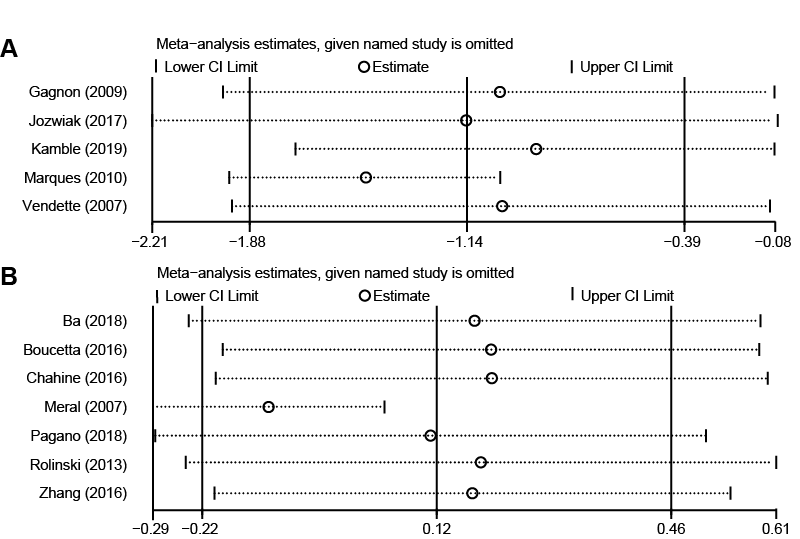


**Figure S5**. Sensitivity analysis of instability in (A) Confirmed-RBD subgroup and (B) Probable-RBD subgroup in generativity studies. CI, Confidence Intervals.


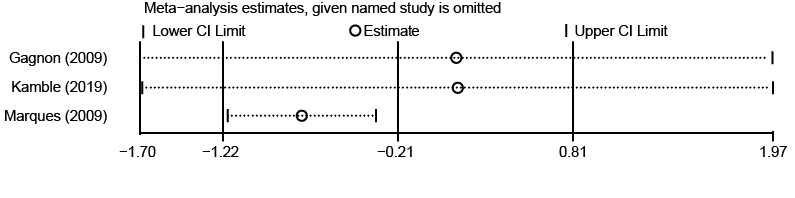


**Figure S6**. Sensitivity analysis of instability in Confirmed-RBD subgroup in updating studies. CI, Confidence Intervals.


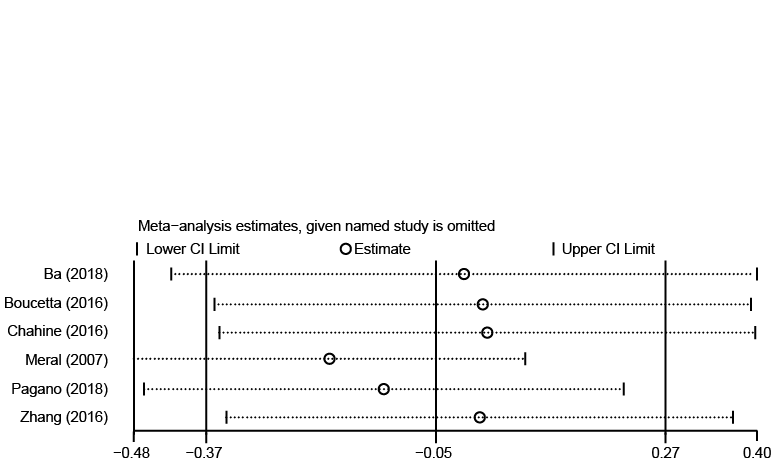


**Figure S7**. Sensitivity analysis of instability in Probable-RBD subgroup in visuospatial/constructional ability studies. CI, Confidence Intervals.


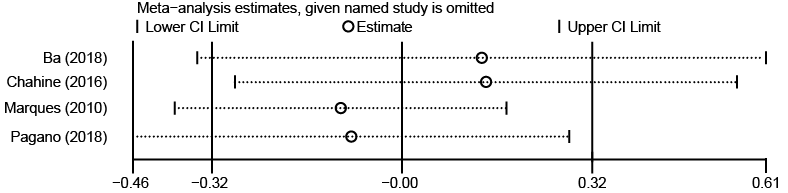


**Figure S8**. Sensitivity analysis of instability in processing speed/complex attention/working memory ability studies. CI, Confidence Intervals.


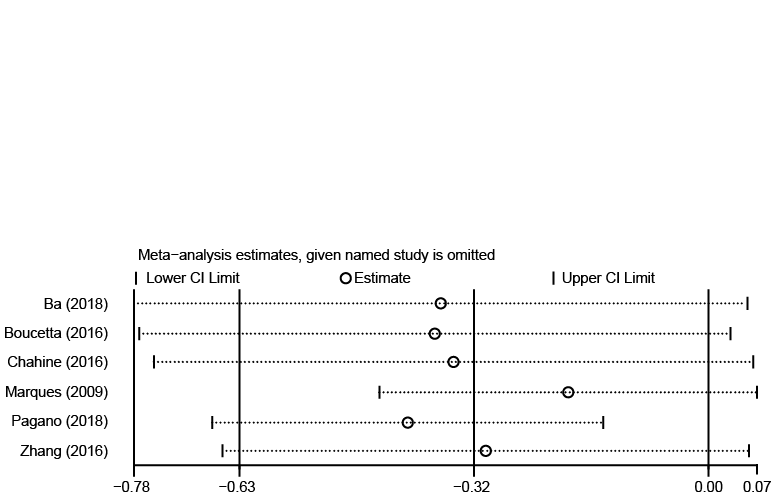


**Figure S9**. Sensitivity analysis of instability in psychomotor ability studies. CI, Confidence Intervals.

Table S1. Search strategy for electronic databases.

| **Embase** | 1. ‘parasomina’/exp 2. ‘rem sleep behavior disorder’:ti,ab 3. ‘rapid eye movement sleep behavior disorder’:ti,ab 4. ‘behavior disorder, rem’:ti,ab 5. ‘behavior disorders, rem’:ti,ab 6. ‘rem behavior disorder’:ti,ab 7. ‘behavior disorder, rapid eye movement sleep’:ti,ab 8. 1 or 2 or 3 or 4 or 5 or 6 or 7 9. ‘cognitive defect’/exp 10. ‘cognitive dysfunction’:ti,ab 11. ‘mild cognitive impairment’:ti,ab 12. ‘cognitive decline’:ti,ab 13. ‘cognitive deficits’:ti,ab 14. ‘cognitive dysfunctions’:ti,ab 15. ‘cognitive impairment’:ti,ab 16. ‘dysfunction, cognitive’:ti,ab 17. ‘dysfunctions, cognitive’:ti,ab 18. ‘cognitive impairments’:ti,ab 19. ‘impairment, cognitive’:ti,ab 20. ‘impairments, cognitive’:ti,ab 21. ‘cognitive impairment, mild’:ti,ab 22. ‘cognitive impairments, mild’:ti,ab 23. ‘impairment, mild cognitive’:ti,ab 24. ‘impairments, mild cognitive’:ti,ab 25. ‘mild cognitive impairments’:ti,ab 26. ‘mild neurocognitive disorder’:ti,ab 27. ‘disorder, mild neurocognitive’:ti,ab 28. ‘disorders, mild neurocognitive’:ti,ab 29. ‘mild neurocognitive disorders’:ti,ab 30. ‘neurocognitive disorder, mild’:ti,ab 31. ‘neurocognitive disorders, mild’:ti,ab 32. ‘cognitive declines’:ti,ab 33. ‘decline, cognitive’:ti,ab 34. ‘declines, cognitive’:ti,ab 35. ‘mental deterioration’:ti,ab 36. ‘deterioration, mental’:ti,ab 37. ‘deteriorations, mental’:ti,ab 38. ‘mental deteriorations’ 39. 9 or 10 or 11 or 12 or 13 or 14 or 15 or 16 or 17 or 18 or 19 or 20 or 21 or 22 or 23 or 24 or 25 or 26 or 27 or 28 or 29 or 30 or 31 or 32 or 33 or 34 or 35 or 36 or 37 or 38 40. ‘parkinson disease’/exp 41. ‘idiopathic parkinson disease’:ti,ab 42. ‘lewy body parkinson disease’:ti,ab 43. ‘primary parkinsonism’:ti,ab 44. ‘parkinsonism’:ti,ab 45. ‘primary parkinson disease’:ti,ab 46. ‘paralysis agitans’:ti,ab 47. ‘parkinson disease, idiopathic’:ti,ab 48. ‘parkinson disease, lewy body’:ti,ab 49. ‘idiopathic parkinson disease’:ti,ab 50. ‘parkinsonism, primary’ 51. 40 or 41 or 42 or 43 or 44 or 45 or 46 or 47 or 48 or 49 or 50 52. 8 AND 39 AND 51 |
| --- | --- |
| **PubMed** | 1. REM Sleep Behavior disorder [Title/Abstract] 2. REM Behavior disorder [Title/Abstract] 3. Rapid Eye Movement Sleep Behavior disorder [Title/Abstract] 4. Behavior disorder, REM [Title/Abstract] 5. Behavior disorders, REM [Title/Abstract] 6. Behavior disorder, Rapid Eye Movement Sleep [Title/Abstract] 7. 1 or 2 or 3 or 4 or 5 or 6 8. Cognitive dysfunction [Title/Abstract] 9. Mild Cognitive Impairment [Title/Abstract] 10. Cognitive decline [Title/Abstract] 11. Cognitive deficits [Title/Abstract] 12. Cognitive dysfunctions [Title/Abstract] 13. Cognitive impairment [Title/Abstract] 14. dysfunction, Cognitive [Title/Abstract] 15. dysfunctions, Cognitive [Title/Abstract] 16. Cognitive Impairments [Title/Abstract] 17. Impairment, Cognitive [Title/Abstract] 18. Impairments, Cognitive [Title/Abstract] 19. Cognitive Impairment, Mild [Title/Abstract] 20. Cognitive Impairments, Mild [Title/Abstract] 21. Impairment, Mild Cognitive [Title/Abstract] 22. Impairments, Mild Cognitive [Title/Abstract] 23. Mild Cognitive Impairments [Title/Abstract] 24. Mild Neurocognitive disorder [Title/Abstract] 25. disorder, Mild Neurocognitive [Title/Abstract] 26. disorders, Mild Neurocognitive [Title/Abstract] 27. Mild Neurocognitive disorders [Title/Abstract] 28. Neurocognitive disorder, Mild [Title/Abstract] 29. Neurocognitive disorders, Mild [Title/Abstract] 30. Cognitive declines [Title/Abstract] 31. decline, Cognitive [Title/Abstract] 32. declines, Cognitive [Title/Abstract] 33. Mental deterioration [Title/Abstract] 34. deterioration, Mental [Title/Abstract] 35. deteriorations, Mental [Title/Abstract] 36. Mental deteriorations [Title/Abstract] 37. 8 or 9 or 10 or 11 or 12 or 13 or 14 or 15 or 16 or 17 or 18 or 19 or 20 or 21 or 22 or 23 or 24 or 25 or 26 or 27 or 28 or 29 or 30 or 31 or 32 or 33 or 34 or 35 or 36 38. parkinson disease [Title/Abstract] 39. parkinson's disease [Title/Abstract] 40. Idiopathic parkinson's disease [Title/Abstract] 41. Lewy Body parkinson disease [Title/Abstract] 42. primary parkinsonism [Title/Abstract] 43. parkinsonism [Title/Abstract] 44. primary parkinson disease [Title/Abstract] 45. paralysis Agitans [Title/Abstract] 46. Lewy Body parkinson's disease [Title/Abstract] 47. parkinson's disease, Idiopathic [Title/Abstract] 48. parkinson's disease, Lewy Body [Title/Abstract] 49. parkinson disease, Idiopathic [Title/Abstract] 50. Idiopathic parkinson disease [Title/Abstract] 51. parkinsonism, primary [Title/Abstract] 52. 38 or 39 or 40 or 41 or 42 or 43 or 44 or 45 or 46 or 47 or 48 or 49 or 50 or 51 53. 7 and 37 and 52 |
| **PROQUEST** | ab(REM Sleep Behavior Disorder) AND ab(Cognitive Dysfunction) AND ab(Parkinson Disease) AND Human |
| **Cocharne** | 1. MeSH descriptor: [REM Sleep Behavior disorder] explode all trees 2. MeSH descriptor: [Cognitive dysfunction] explode all trees 3. MeSH descriptor: [Parkinson disease] explode all trees 4. (REM Behavior disorder):ti,ab,kw 5. (Rapid Eye Movement Sleep Behavior disorder):ti,ab,kw 6. (Behavior disorder, REM):ti,ab,kw 7. (Behavior disorders, REM):ti,ab,kw 8. (Behavior disorder, Rapid Eye Movement Sleep):ti,ab,kw 9. 1 or 4 or 5 or 6 or 7 or 8 10. (Mild Cognitive Impairment):ti,ab,kw 11. (Cognitive decline):ti,ab,kw 12. (Cognitive deficits):ti,ab,kw 13. (Cognitive dysfunctions):ti,ab,kw 14. (Cognitive impairment):ti,ab,kw 15. (dysfunction, Cognitive):ti,ab,kw 16. (dysfunctions, Cognitive):ti,ab,kw 17. (Cognitive Impairments):ti,ab,kw 18. (Impairment, Cognitive):ti,ab,kw 19. (Impairments, Cognitive):ti,ab,kw 20. (Cognitive Impairment, Mild):ti,ab,kw 21. (Cognitive Impairments, Mild):ti,ab,kw 22. (Impairment, Mild Cognitive):ti,ab,kw 23. (Impairments, Mild Cognitive):ti,ab,kw 24. (Mild Cognitive Impairments):ti,ab,kw 25. (Mild Neurocognitive disorder):ti,ab,kw 26. (disorder, Mild Neurocognitive):ti,ab,kw 27. (disorders, Mild Neurocognitive):ti,ab,kw 28. (Mild Neurocognitive disorders):ti,ab,kw 29. (Neurocognitive disorder, Mild):ti,ab,kw 30. (Neurocognitive disorders, Mild):ti,ab,kw 31. (Cognitive declines):ti,ab,kw 32. (decline, Cognitive):ti,ab,kw 33. (declines, Cognitive):ti,ab,kw 34. (Mental deterioration):ti,ab,kw 35. (deterioration, Mental):ti,ab,kw 36. (deteriorations, Mental):ti,ab,kw 37. (Mental deteriorations):ti,ab,kw 38. 2 or 10 or 11 or 12 or 13 or 14 or 15 or 16 or 17 or 18 or 19 or 20 or 21 or 22 or 23 or 24 or 25 or 26 or 27 or 28 or 29 or 30 or 31 or 32 or 33 or 34 or 35 or 36 or 37 39. (parkinson disease):ti,ab,kw 40. (parkinson's disease):ti,ab,kw 41. (Idiopathic parkinson's disease):ti,ab,kw 42. (Lewy Body parkinson disease):ti,ab,kw 43. (primary parkinsonism):ti,ab,kw 44. (parkinsonism):ti,ab,kw 45. (primary parkinson disease):ti,ab,kw 46. (paralysis Agitans):ti,ab,kw 47. (Lewy Body parkinson's disease):ti,ab,kw 48. (parkinson's disease, Idiopathic):ti,ab,kw 49. (parkinson's disease, Lewy Body):ti,ab,kw 50. (parkinson disease, Idiopathic):ti,ab,kw 51. (Idiopathic parkinson disease):ti,ab,kw 52. (parkinsonism, primary):ti,ab,kw 53. 2 or 39 or 40 or 41 or 42 or 43 or 44 or 45 or 46 or 47 or 48 or 49 or 50 or 51 or 52 54. 9 AND 38 AND 53 |

Table S2. Modified Newcastle-Ottawa Scale for risk of bias assessment.

| Case Control Studies | Cohort Studies | Cross-sectional Studies |
| --- | --- | --- |
| Selection:  1. Is the case (PD-RBD patients) definition adequate?  a) yes, with independent validation☆  b) yes, e.g., record linkage or based on self-reports  c) no description  2. Representativeness of the cases (PD-RBD patients)  a) consecutive or obviously representative series of cases☆  b) potential for selection biases or not stated  3. Selection of Controls (PD-NRBD patients)  a) community controls☆  b) hospital controls  c) no description  4. Definition of Controls (PD-NRBD patients)  a) no history of disease (endpoint) ☆  b) no description of source | Selection:  1. Representativeness of the exposed cohort  a) truly representative of the average PD-RBD patients in the community☆  b) somewhat representative of the average PD-RBD patients in the community☆  c) selected group of users e.g., nurses, volunteers  d) no description of the derivation of the cohort  2. Selection of the non-exposed cohort (PD-NRBD patients)  a) drawn from the same community as the exposed cohort☆  b) drawn from a different source  c) no description of the derivation of the non-exposed cohort  3. Ascertainment of exposure  a) secure record (e.g., PSG records) ☆  b) structured interview☆  c) written self-report  d) no description  4. Demonstration that outcome of interest was not present at study start  a) yes☆  b) no | Selection:  1. Representativeness of the exposed cohort (PD-RBD patients)  a) truly representative of the average in the target population (all subjects or random sampling) ☆  b) somewhat representative of the average in the target population (non-random sampling) ☆  c) selected group of users  d) no description of the sampling strategy  2. Sample size  a) justified and satisfactory☆  b) not justified  3. Non-respondents  a) comparability between respondents and non-respondents characteristics is established, and the response rate is satisfactory☆  b) the response rate is unsatisfactory, or the comparability between respondents and non-respondents is unsatisfactory  c) no description of the response rate or the characteristics of the responders and the non-responders  4. Ascertainment of the exposure  a) validated measurement tool☆☆  b) non-validated measurement tool, but the tool is available or described☆  c) no description of the measurement tool. |
| Comparability:  1. Comparability of cases and controls on the basis of the design or analysis  a) study controls for cognitive status☆  b) study controls for any additional factor (e.g., age, gender, severity of illness) ☆ | Comparability:  1. Comparability of cohorts on the basis of the design or analysis  a) study controls for cognitive status☆  b) study controls for any additional factor (e.g., age, gender, severity of illness) ☆ | Comparability:  1. The subjects in different outcome groups are comparable, based on the study design or analysis. Confounding factors are controlled.  a) the study controls for the most important factor (cognitive status) ☆  b) the study control for any additional factor (e.g., age, gender, severity of illness) ☆ |
| Exposure  1. Ascertainment of exposure  a) secure record (e.g., surgical records) ☆  b) structured interview where blind to case/control status☆  c) interview not blinded to case/control status  d) written self-report or medical record only  e) no description  2. Same method of ascertainment for cases and controls  a) yes☆  b) no  3. Non-Response rate  a) same rate for both groups☆  b) non respondents described  c) rate different and no designation | Outcome  1. Assessment of outcome  a) independent blind assessment☆  b) record linkage☆  c) self-report  d) no description  2. Was follow-up long enough for outcomes to occur  a) yes☆  b) no  3. Adequacy of follow up of cohorts  a) complete follow up - all subjects accounted for☆  b) subjects lost to follow up unlikely to introduce bias: i.e., <10% small number, lost, or description provided of those lost☆  c) follow-up rate < 90% and no description of those lost  d) no statement | Outcome  1. Assessment of outcome  a) independent blind assessment☆☆  b) record linkage☆☆  c) self-report☆  d) no description  2. Statistical test  a) the statistical test used to analyze the data is clearly described and appropriate, and the measurement of the association is presented, including confidence intervals and the probability level (p value) ☆  b) the statistical test is not appropriate, not described or incomplete |

Table S3a. Quality assessment scores for included case control studies.

| Study | Selection | | | | Comparability | Exposure | | | Total |
| --- | --- | --- | --- | --- | --- | --- | --- | --- | --- |
|  | 1 | 2 | 3 | 4 | 1 | 1 | 2 | 3 |  |
| Arnaldi 2015 | ☆ | ☆ | ☆ | ☆ | ☆☆ | ☆ | ☆ |  | 8 |
| Boucetta 2016 | ☆ |  | ☆ | ☆ | ☆☆ | ☆ | ☆ |  | 7 |
| Gagnon 2004 | ☆ | ☆ | ☆ | ☆ | ☆☆ | ☆ | ☆ |  | 8 |
| Gagnon 2009 | ☆ |  | ☆ | ☆ | ☆☆ | ☆ | ☆ |  | 7 |
| Gaudreault 2013 | ☆ | ☆ | ☆ | ☆ | ☆☆ | ☆ | ☆ |  | 8 |
| Jozwiak 2017 | ☆ | ☆ | ☆ | ☆ | ☆☆ | ☆ | ☆ |  | 8 |
| Kamble2019 | ☆ | ☆ | ☆ | ☆ | ☆ | ☆ | ☆ |  | 7 |
| Kim 2016 | ☆ |  | ☆ | ☆ | ☆☆ | ☆ | ☆ |  | 7 |
| Kotagal 2012 | ☆ |  | ☆ | ☆ | ☆ | ☆ | ☆ |  | 6 |
| Lim 2016 | ☆ | ☆ | ☆ | ☆ | ☆☆ | ☆ | ☆ |  | 8 |
| Marques 2010 | ☆ |  | ☆ | ☆ | ☆☆ | ☆ | ☆ |  | 7 |
| Nardone 2013 | ☆ |  |  | ☆ | ☆☆ | ☆ | ☆ |  | 6 |
| Plomhause 2014 | ☆ |  |  | ☆ | ☆☆ | ☆ | ☆ |  | 6 |
| Rahmani 2016 | ☆ |  |  | ☆ | ☆☆ | ☆ | ☆ |  | 6 |
| Sinforiani 2006 | ☆ |  | ☆ | ☆ | ☆☆ | ☆ | ☆ |  | 7 |

Table S3b. Quality assessment scores for included cohort studies.

| Study | Selection | | | | Comparability | Outcome | | | Total |
| --- | --- | --- | --- | --- | --- | --- | --- | --- | --- |
|  | 1 | 2 | 3 | 4 | 1 | 1 | 2 | 3 |  |
| Ba 2018 |  | ☆ | ☆ |  | ☆☆ | ☆ | ☆ | ☆ | 8 |
| Chahine 2016 |  | ☆ | ☆ | ☆ | ☆☆ | ☆ | ☆ | ☆ | 8 |
| Duarte Folle 2019 | ☆ | ☆ | ☆ | ☆ | ☆☆ | ☆ | ☆ | ☆ | 9 |
| Gjerstad 2008 | ☆ | ☆ | ☆ | ☆ | ☆ | ☆ | ☆ | ☆ | 8 |
| Lavault 2010 | ☆ | ☆ | ☆ | ☆ | ☆☆ | ☆ | ☆ | ☆ | 9 |
| Nomura 2013 | ☆ | ☆ | ☆ | ☆ | ☆☆ | ☆ | ☆ | ☆ | 9 |
| Pagano 2018 |  | ☆ | ☆ | ☆ | ☆☆ | ☆ | ☆ | ☆ | 8 |
| Postuma 2012 |  | ☆ | ☆ | ☆ | ☆☆ | ☆ | ☆ | ☆ | 8 |

Table S3c. Quality assessment scores for included cross-sectional studies.

| Study | Selection | | | | Comparability | Outcome | | Total |
| --- | --- | --- | --- | --- | --- | --- | --- | --- |
|  | 1 | 2 | 3 | 4 | 1 | 1 | 2 |  |
| Ford 2013 |  |  |  | ☆ | ☆☆ | ☆☆ | ☆ | 6 |
| Huang 2018 |  | ☆ |  | ☆ | ☆☆ | ☆☆ | ☆ | 7 |
| Kim 2014 | ☆ | ☆ |  | ☆ | ☆ | ☆☆ | ☆ | 7 |
| Lee 2010 | ☆ | ☆ |  | ☆ | ☆ | ☆☆ | ☆ | 7 |
| Liu 2019 | ☆ | ☆ |  | ☆ | ☆☆ | ☆☆ | ☆ | 8 |
| Mahale 2016 | ☆ |  |  | ☆ | ☆ | ☆☆ | ☆ | 6 |
| Meral 2007 | ☆ |  |  | ☆ | ☆☆ | ☆☆ | ☆ | 7 |
| Naismith 2011 |  |  |  | ☆ | ☆☆ | ☆☆ | ☆ | 6 |
| Nomura 2010 |  |  |  | ☆ | ☆☆ | ☆☆ | ☆ | 6 |
| Nomura 2016 |  |  |  | ☆ | ☆☆ | ☆☆ | ☆ | 6 |
| Nomura 2017 | ☆ |  |  | ☆ | ☆☆ | ☆☆ | ☆ | 7 |
| Plomhause 2013 | ☆ |  | ☆ | ☆ | ☆☆ | ☆☆ | ☆ | 8 |
| Rolinski 2014 | ☆ | ☆ |  | ☆ | ☆☆ | ☆☆ | ☆ | 8 |
| Sixel-doring 2011 | ☆ | ☆ |  | ☆ | ☆ | ☆☆ | ☆ | 7 |
| Vendette 2007 |  |  |  | ☆ | ☆☆ | ☆☆ | ☆ | 6 |
| Zhang 2016 |  |  |  | ☆ | ☆☆ | ☆☆ | ☆ | 6 |

Newcastle-Ottawa question numbers are detailed in Table S4. Minimum number of possible stars to be awarded = 0, maximum number of possible stars to be awarded = 9.

Table S4. Meta-regression analysis for effect of RBD on cognitive damages in patients with PD.

|  | | Age at evaluation | Gender | Edu | PD duration | UPDRS-III | H&Y stage | LEDD | Cognitive test | RBD assessment |
| --- | --- | --- | --- | --- | --- | --- | --- | --- | --- | --- |
| Global Cognitive Function | K | 38 | 35 | 20 | 36 | 28 | 24 | 33 | 38 | 38 |
|  | β | -0.019 | -0.277 | 0.030 | -0.026 | -0.002 | -0.089 | 0.000 | 0.221 | 0.166 |
|  | p | 0.270 | 0.576 | 0.315 | 0.261 | 0.908 | 0.536 | 0.529 | **0.041** | 0.238 |
| Memory-Long Term Verbal Recall | K | 11 | 9 | 7 | 11 | 8 | 7 | 11 | 11 | 11 |
|  | β | 0.008 | -0.011 | -0.156 | -0.059 | 0.002 | -0.001 | 0.000 | 0.135 | 0.451 |
|  | p | 0.254 | 0.084 | 0.061 | 0.054 | 0.893 | 0.350 | 0.601 | **0.039** | 0.131 |
| Memory-Long Term Verbal Recognition | K | 8 | 6 | 6 | 8 | 6 | 6 | 8 | 8 | 8 |
|  | β | 0.040 | -2.183 | 0.028 | 0.000 | 0.027 | -0.594 | 0.000 | 0.336 | 0.549 |
|  | p | 0.604 | 0.791 | 0.611 | 0.991 | 0.572 | 0.055 | 0.860 | **0.000** | 0.066 |
| EF-Generativity | K | 12 | 9 | 8 | 12 | 9 | 7 | 12 | - | 12 |
|  | β | 0.096 | -2.120 | -0.084 | -0.050 | -0.057 | -1.385 | -0.001 | - | 1.294 |
|  | p | 0.418 | 0.468 | 0.392 | 0.666 | 0.391 | 0.074 | 0.453 | - | **0.013** |
| EF-Inhibition | K | 7 | - | - | 7 | - | - | 7 | 7 | 7 |
|  | β | 0.078 | - | - | 0.157 | - | - | 0.000 | 0.558 | -0.264 |
|  | p | 0.103 | - | - | 0.287 | - | - | 0.991 | **0.011** | 0.462 |
| EF-Shifting | K | 7 | - | - | 7 | - | 6 | 7 | 7 | 7 |
|  | β | -0.012 | - | - | 0.004 | - | 0.001 | 0.000 | -0.164 | -0.417 |
|  | p | 0.890 | - | - | 0.940 | - | 0.996 | 0.791 | 0.099 | **0.033** |
| EF-Updating | K | 6 | - | - | 6 | - | - | 6 | - | 6 |
|  | β | -0.053 | - | - | 0.157 | - | - | 0.000 | - | 0.044 |
|  | p | 0.621 | - | - | 0.496 | - | - | 0.898 | - | 0.937 |
| Visuospatial/ Constructional Ability | K | 9 | 7 | 7 | 9 | 7 | 6 | 9 | 9 | 9 |
|  | β | 0.035 | -0.995 | 0.035 | 0.000 | 0.036 | -0.835 | 0.000 | -0.576 | 0.575 |
|  | p | 0.654 | 0.905 | 0.598 | 0.997 | 0.719 | **0.033** | 0.811 | **0.048** | 0.080 |
| Psychomotor Ability | K | 6 | 6 | 6 | 6 | 5 | - | 6 | - | 6 |
|  | β | -0.009 | 6.310 | 0.155 | -0.225 | 0.065 | - | -0.003 | - | 2.239 |
|  | p | 0.982 | **0.001** | 0.095 | **0.005** | 0.831 | - | **0.005** | - | **0.001** |

Edu = Education; UPDRS III = Unified Parkinson’s Disease Rating Scale, part 3; H&Y stage = Hoehn & Yahr stage; LEDD = Levodopa Equivalent Daily Dose; K = number of studies; EF = executive function; Statistically significant values are reported in bold.
